# Identifying core adverse health outcomes for frailty assessment in older adults using administrative data

**DOI:** 10.3389/fmed.2025.1678317

**Published:** 2025-12-15

**Authors:** Margherita Silan, Maurizio Nicolaio, Erika Banzato, Giovanna Boccuzzo

**Affiliations:** Department of Statistical Sciences, University of Padova, Padova, Italy

**Keywords:** administrative healthcare data, adverse health outcomes, aging, factor analysis, frailty, graphical models, outcome selection

## Abstract

**Objectives:**

Measurement of frailty can be based on the ability to predict adverse health outcomes. Although frailty research is progressing rapidly, a unique work that analyzes together outcomes related to frailty condition is still lacking in literature. This article aims to fill this gap, selecting a parsimonious set of outcomes relevant in frailty studies that exploit administrative healthcare data.

**Methods:**

Starting with an extensive literature review, we identified several health outcomes that can be measured with administrative healthcare databases. We computed the prevalence and correlation of these outcomes in a local health unit in North-East Italy. We performed a factor analysis and estimated a graphical model to examine the conditional independence relationships between the outcomes.

**Results:**

Our analysis revealed two primary outcome groups: adverse events (characterized by various forms of hospital use) and adverse conditions (such as dementia and disability). Femur fracture emerged as a distinct outcome, while death showed positive associations with all other outcomes. Considering overlaps and relationships, we selected a core set of six representative outcomes: death, high priority access to the emergency room, femur fracture, hospitalization, disability, and dementia.

**Conclusion:**

This study identified six central and non-redundant adverse health outcomes related to frailty that can be easily derived from routinely available administrative healthcare data. These findings provide a methodologically grounded selection of outcomes that are clinically meaningful and feasible, offering a solid foundation for developing population-based frailty indices.

## 1 Introduction

The global demographic landscape is undergoing a significant change, with the number and proportion of older adults increasing rapidly. According to the World Health Organization (1), for the first time in history, the number of people 60 years and older surpassed the number of children under 5 years of age in 2020, and this trend is expected to continue.

Italy has the highest proportion of older adults in Europe. This is the result of rising life expectancy and falling birth rates: of 58.9 million residents, 24.1% are aged 65 or above (2).

Individuals of the same chronological age can differ considerably in their health status. As people grow older, their resilience to adverse events varies due to underlying differences in their physiological and biological condition. These differences are influenced by lifestyle factors such as diet, exercise, smoking and stress; environmental factors such as pollution and climate change; and social factors such as loneliness, social support and socioeconomic status (3–5).

The concept of frailty was first introduced in Vaupel’s seminal work, whose objective was to account for the heterogeneity of mortality rates between individuals of the same chronological age, under the assumption that individuals have varying levels of susceptibility to adverse health outcomes (6). Such susceptibility has been conceptualized as frailty: an underlying unmeasured variable affecting population-level mortality patterns. This work laid the groundwork for the emergence of an important strand of scientific research focused on aging and frailty. Such interest has been fueled over time by population aging processes, calling for the attempt to define and measure frailty and understand its relations with adverse health outcomes at older ages. Since then, researchers have focused on identifying frail individuals and predicting their risk of adverse health outcomes, to develop tailored interventions and care plans.

Even if frailty is now a widely used term, a unique definition of frailty is still lacking in literature. The concept describes a complex and multidimensional individual condition, and experts have proposed different definitions depending on their area of interest. Indeed, the concept of frailty is constantly evolving in the literature, and there is a progressive debate on how to define the condition (7, 8). However, there are two fundamental aspects that recur in scientific articles dealing with this topic: frailty as a complex and multidimensional condition, involving multiple functional domains; and frailty as a state of susceptibility to adverse health outcomes (9).

A better understanding of frailty can lead to interventions to reduce its prevalence and impact (10). Therefore, measuring frailty is an indispensable task to provide appropriate and timely care. This requires linking the conceptual definition of frailty with an operational one. The operational definition must be based on applicable criteria derived from available data and must specify in a clear and measurable way the criteria to be followed to determine the existence of frailty and its measurement (9).

Rockwood (11) identified three basic criteria for an effective definition of frailty: content validity (whether the definition makes sense on first principles), construct validity (whether the operational definition coheres with other measures of the phenomenon, related conditions, and constructs), and criterion validity (when a new definition or test correctly classifies people based on a referent outcome). According to the criterion validity, the definition must correctly classify individuals against adverse health outcomes.

From an operational point of view, the criterion validity is the reference to identify measures of frailty. It consists of identifying a frailty index that allows subjects to be classified according to one or more outcomes that are considered consequences of frailty. Some commonly considered outcomes include death, institutionalization, hospitalization, and falls.

The process consists of the following steps:

1. Identify the outcome(s) that the frailty index should accurately predict.
2. Identify the variables that make up the frailty index using the predictive approach. If only one outcome is considered, it is fairly simple to proceed, for example, using regression approaches that consider the outcome as the response variable and the variables explaining the outcome as explanatory variables, which will make up the frailty index. If there are more than one outcome, it is necessary to adopt more sophisticated statistical techniques that identify the subset of variables that best predicts multiple outcomes simultaneously.
3. Identify the method for calculating the index. In the case of a single outcome, the frailty index can be a weighted average of the explanatory variables, with weights given by the regression coefficients [see, e.g., (12)]. In the case of multiple outcomes, once the core variables that best predict them have been identified, an approach for constructing the indicator must be defined from among those already known in composite index theory [see, e.g., (13)].

Although the predictive approach appears to be the driving point toward an operative definition of frailty, there is no clear or consistent agreement in the literature on which health outcomes should be used to assess it.

The choice of outcomes depends on the objectives of the study and the available data: the National Health System is increasingly focusing on stratifying the entire population according to its level of frailty, multimorbidity, or health needs, to optimize increasingly scarce resources. In this context, it is necessary to refer to data covering the entire population, i.e., administrative data. With administrative data some outcomes (e.g., death, hospitalization) will be observable, but not others that are not recorded (e.g., functional impairment). If the objective is not to stratify the entire population, but, for instance, to study frailty from several points of view (health, social, cognitive), it is possible to refer to sample data sources, which typically provide information on perceived health, functional abilities, or other outcomes.

In this paper, we focus on a study of frailty outcomes in the context of the national health system and the need to stratify the entire population, for which we will refer to administrative health data sources.

To the best of our knowledge, no single study has simultaneously examined and analyzed multiple outcomes associated with frailty. To address this gap, the aim of the present work is to investigate the adverse health outcomes related to frailty. Specifically, the objectives are:

1. To conduct a comprehensive literature review to identify which health outcomes related to frailty are most frequently considered in a predictive approach.
2. To estimate the extent to which these outcomes are used to construct frailty indices.
3. To study the occurrence of these health outcomes and the correlation among them using administrative data.
4. To propose a core set of adverse health outcomes related to frailty in the context of health administrative data.

The article is organized as follows: Section 2.1 presents a review of the literature to identify a list of frailty-related outcomes; Section 2.2 describes the administrative health data used in this study; Section 2.3 defines and justifies ten selected outcomes operationalized using administrative health data. Section 2.4 explains the methods used in the statistical analysis. Section 3 reports all results, including: the occurrence of the outcomes (3.1); patterns and correlations among the selected outcomes (3.2); and the analysis of the interrelationships among them (3.3). The discussion and conclusions propose and justify a core set of frailty outcomes (Sections 4 and 5).

## 2 Materials and methods

### 2.1 Literature review

#### 2.1.1 The measurement of frailty based on predictive ability

Many frailty measures rely on the ability to predict adverse health outcomes in the older population (14–16), primarily death, but also other events such as hospital admissions, emergency hospitalizations, institutionalization. In summary, the approach involves the use of statistical models (such as logistic regression or other more advanced statistical techniques) to identify the most relevant variables, including demographic characteristics, clinical conditions, and utilization of healthcare services, that are capable of predict health outcomes.

The results of these models allow for the selection of the most predictive variables, from which a frailty index is then constructed, using various approaches described in the literature, such as a weighted average, a simple sum, or other aggregation methods (15–18). At this stage, each individual is assigned a frailty score that represents his or her risk of experiencing one or more adverse health outcomes.

The literature on this topic is extensive (19, 20). Many studies consider one or, at most, two outcomes (21–24), while others consider the number of events, and some others consider more than two outcomes simultaneously (16, 17). Although statistical approaches vary, the underlying logic remains largely consistent.

Whether based on administrative data or *ad hoc* surveys, it is evident that the choice of adverse outcomes to be considered influences the subsequent steps of the analysis: omitting certain outcomes may lead to the exclusion of relevant variables in the construction of the frailty index; conversely, including outcomes that are too closely correlated may result in an overestimation of the role of some variables.

#### 2.1.2 Outcomes related to frailty conditions

In the literature, different sets of outcomes are considered to detect frailty, depending on the scope and focus of the research. However, there is a lack of consensus on a standardized set of outcomes for frailty assessment. Researchers often select their own set of outcomes without a systematic verification or comparison with other studies. To address this variability and identify the diverse sets of outcomes used to detect frailty across different studies, a careful review of the literature was conducted. We did not perform a single systematic review because the literature is highly heterogeneous and based on articles with different objectives, some of a more conceptual nature, and many others aimed at producing measures of frailty. Since different outcomes can be identified depending on whether the data source is administrative or sample-based, we organized the bibliographic research using the data source criterion.

**Figure 1:**
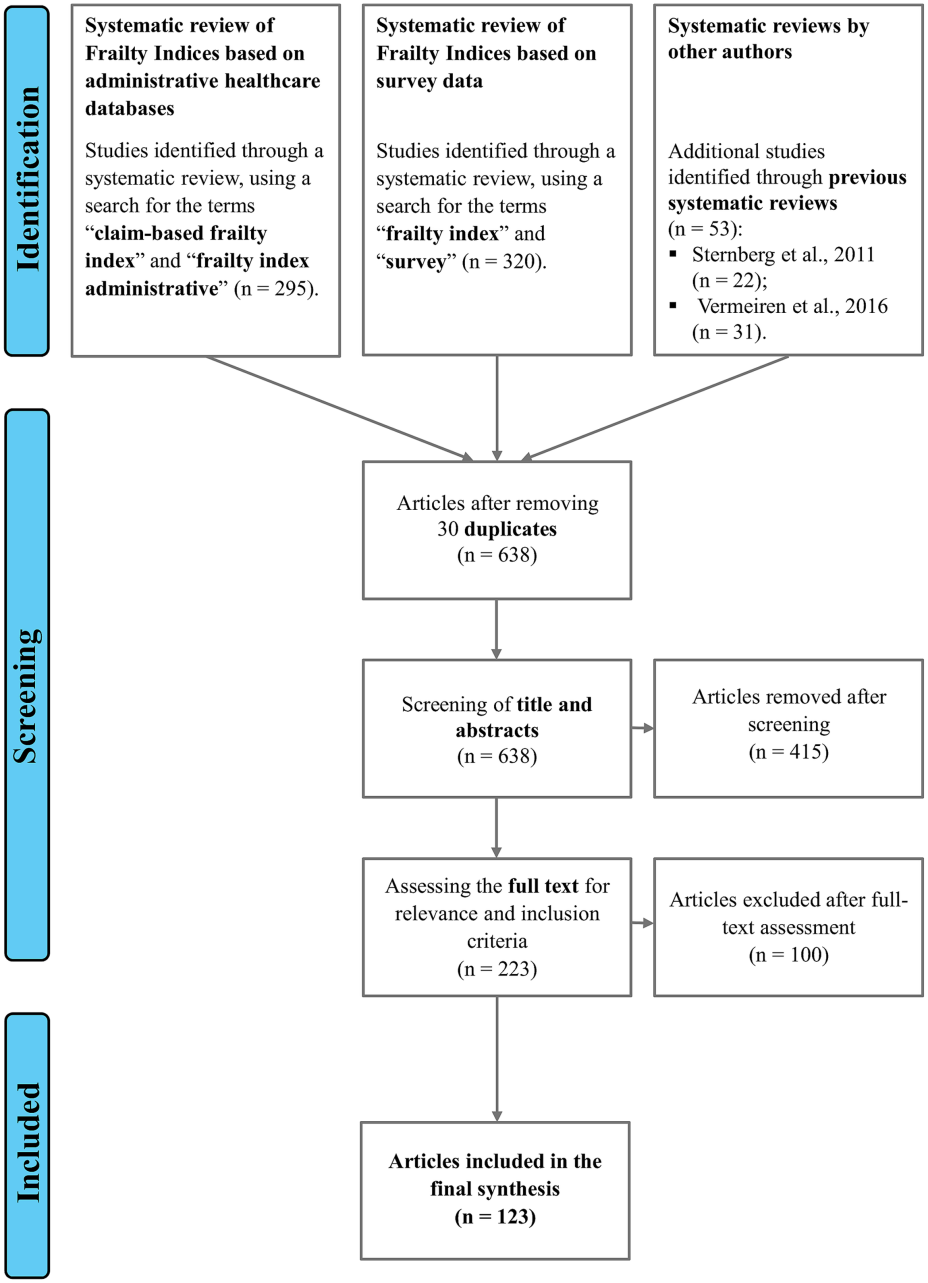
Summary diagram of the literature review.

The results presented here are based on three distinct sources: two targeted systematic reviews and a set of additional articles identified through previously published systematic reviews by other authors (19, 20) (Figure 1). The two systematic reviews were conducted in parallel using the PubMed database during April 2024, one focusing on studies using administrative data and the other on studies based on survey data, representing the two main streams of literature on frailty measures. The first review aimed to identify frailty measures based on administrative data. It yielded 295 articles using the search terms “Claim-based frailty index” and “frailty index administrative.” Of these, 205 were identified through the PubMed database, 62 were selected from two systematic reviews conducted by Lim et al. (25) and Shashikumar et al. (26), and an additional 28 articles were identified through citations from previously analyzed studies. The second review focused on identifying frailty measures derived from population surveys. It returned 320 articles using the keywords “frailty index” AND “survey.” Of these, 272 were identified through the PubMed database, while the remaining 48 were identified through citations of studies previously collected and studied.

Additionally, 53 more articles were selected by reviewing the references from a major systematic review by Sternberg et al. (19) and a meta-analysis by Vermeiren et al. (20). In Sternberg et al. (19), the authors investigated clinical definition, screening tools, and outcomes of frailty. The Medline database was queried through a search string that produced 4,334 articles published between 1997 and 2009 in output. Studies were selected for inclusion if they examined adult patient populations living in the community older than 65 years and provided clinically relevant outcomes, such as hospitalization, death, or change in functional status. After the review process, 22 publications were included. Vermeiren et al. (20) performed a systematic review and meta-analysis examining the predictive value of frailty for negative health outcomes in older adults. After a detailed literature search on PubMed, Web of Knowledge, and PsycInfo (last search in January 2016), the initial search yielded 1,694 articles. After selecting the title and abstract, 291 articles remained for full-text analysis. The selection process was carried out independently by two reviewers that included 31 articles for systematic review and meta-analysis, after excluding 255 according to content and methodological quality criteria.

The initial search yielded 668 articles in total, then they were screened by two reviewers independently under the supervision of a third researcher. After removing 30 duplicates, the titles and abstracts of 638 articles were screened. Of these, 415 did not meet the eligibility criteria and were excluded. Specifically, excluded studies relied exclusively on data derived from specific clinical data sets or from biomedical, physical, or laboratory tests; assessed frailty based only on clinical judgment alone; were duplicates carried out by the same author on the same data set at different time points; assessed the association between outcomes and comorbidity, disability, or vulnerability, but not frailty itself; or did not examine the relationship between outcomes and frailty. The full texts of the remaining 223 articles were evaluated and 100 were subsequently excluded because they only replicate existing frailty measures on different data. As a result, 123 studies were included in the final analysis. Our literature review and selection process adhered to the PRISMA 2020 guidelines [Preferred Reporting Items for Systematic Reviews and Meta-Analyzes; Page et al. (27)]. This approach ensured a rigorous and scientifically standardized review of literature on this topic. However, the primary purpose of our review was to identify a set of health outcomes associated with the frailty condition, rather than to estimate any specific effect.^1^

**Figure 2:**
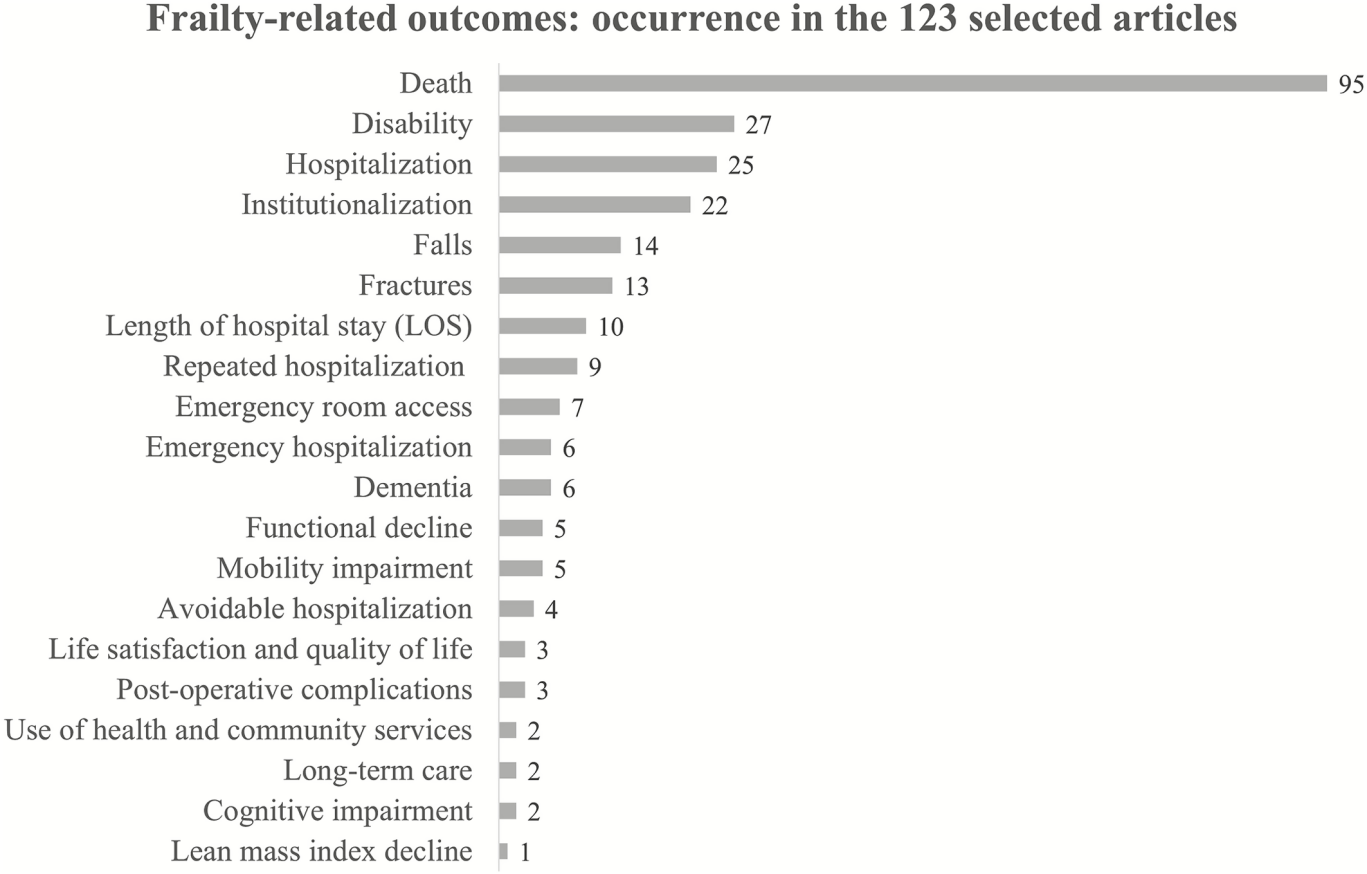
Number of articles for each frailty-related outcome among the 123 selected scientific papers.

Figure 2 summarizes the results of the literature review showing how many of the 123 considered publications were citing the investigated outcomes. Death stands out significantly with 95 citations, far exceeding any other health outcome. Disability is the second most cited outcome with 27 citations of 123 articles, while hospitalization and institutionalization follow with 25 and 22 citations, respectively. Approximately one-tenth of the articles consulted identified falls and fractures as health outcomes related to frailty, with 14 and 13 citations, respectively. Other outcomes such as length of hospital stay, repeated and emergency hospitalization, emergency room access, and dementia have fewer citations, ranging from 6 to 10. The number of citations represent a proxy to gauge how widely recognized an outcome is as being associated with frailty in the scientific community.

In the following sections, the most frequently cited outcomes are described and explained in more detail.

#### 2.1.3 Death

Death represents the last outcome of a series of harmful changes to the body, leading to a progressive decline in the functionality of organs and systems until the “irreversible cessation of brain function” (28, 29). According to the National Institute of Statistics (30), the main causes of death in the older population (aged 65 years and over) in 2022 were cardiovascular diseases, malignant neoplasms, COVID-19, and respiratory system diseases.

#### 2.1.4 Disability

Disability was first defined in the International Classification of Impairments, Disabilities and Handicaps (ICIDH) system in 1980 (31) as “any limitation or loss (resulting from an impairment) of the ability to perform an activity in the manner or within the range considered normal for a human being.” In other words, the WHO’s ICIDH defines disability as difficulty or dependence in performing essential daily living and self-care activities. It can manifest socially (inability to communicate or leave home for physical or mental reasons) or physically (mobility problems). Disability is usually measured using questionnaires or scales, such as the ADL (Activities of Daily Living) and IADL (Instrumental Activities of Daily Living) scales. The ADL scale identifies limitations in basic daily activities necessary for survival and self-sufficiency (32). The IADL scale refers to activities requiring greater adaptation and independence, such as shopping, cooking, cleaning, managing money, and using the telephone (33). Although the ICIDH represented the first systematic attempt to classify disability, it was later replaced by more comprehensive frameworks. In particular, the International Classification of Functioning, Disability and Health (ICF), introduced by the WHO in 2001, conceptualized disability as the result of complex interactions between health conditions and contextual environmental and personal factors. Despite this conceptual evolution, most large-scale statistical surveys have continued to rely on measurement tools rooted in the earlier ICIDH model. These instruments—more linear and easier to operationalize in population studies—remain the basis for assessing limitations in daily functioning, even though they capture a more restricted view of disability compared with the multidimensional ICF framework. Recent data from the Italian National Institute of Statistics show that 7.1% of people aged 65–74 years have severe limitations in daily activities, rising to 20.4% among those aged 75 and older, with lower rates than a decade earlier (34).

#### 2.1.5 Hospitalization

Hospitalization is a key outcome of frailty in older adults, often indicating a critical decline in health and independence. Frail older adults are more susceptible to acute illnesses, falls, and exacerbations of chronic diseases, which often lead to increased hospital admissions (35). These hospitalizations can result in further functional decline, complications, and a higher risk of subsequent readmissions, creating a cycle that underscores the vulnerability of frail older adults in healthcare settings (36). Furthermore, frailty substantially increases the risk of urgent hospitalization among older adults (37). These urgent hospitalizations often result from acute exacerbations of chronic conditions, falls, or infections, to which frail older adults are particularly susceptible (35). Recognizing frailty as a risk factor for urgent hospitalization is crucial to developing targeted interventions to prevent such events and improve outcomes for this vulnerable population (38). Moreover, frailty has been shown to prolong hospital stay, which is likely to be associated with increased cost of treatment (39). Additionally, many chronic or acute diseases can be managed through primary care, allowing healthcare resources to be reserved for true emergencies (40). “Avoidable hospitalization” refers to hospitalizations that can be prevented by timely and effective primary and ambulatory care. This outcome helps identify vulnerable individuals for whom primary care is suboptimal (16). Conditions leading to avoidable hospitalizations are known as Ambulatory Care Sensitive Conditions (ACSC), divided into acute events (e.g., nutritional deficiencies, dehydration) and chronic events (e.g., diabetes, hypertension). Rosano et al. (40) adapted the definition of avoidable hospitalization to the Italian context, identifying specific diagnoses from hospital discharge records. Repeated hospitalizations, often due to worsening conditions after discharge, affect the management of healthcare resources, leading to increased bed occupancy and financial pressure on the healthcare system. In conclusion, the impact of frailty extends beyond initial hospitalization, as frail patients typically experience longer hospital stays, higher rates of complications, and a greater likelihood of readmission (41). Moreover, it is worth noting that all these events share a common characteristic: they are health outcomes that are not solely dependent on the individual but are also mediated by healthcare organizations. This includes not only hospital-based services, but also the broader network of district healthcare and community-based care systems. As such, these outcomes reflect both the individual’s health status and the effectiveness and accessibility of the entire healthcare system in managing frailty.

#### 2.1.6 Institutionalization

Severe disability is often associated with institutionalization in nursing homes, a major outcome of frailty in older adults, often viewed as a last resort when independent living becomes difficult. Although not all old frail individuals are institutionalized, it is rare for healthy older adults to require such care. Various medical and social factors contribute to the risk of institutionalization (42). Indeed, the decision to move an older person into institutional care is complex, involving both the individual’s health status and their caregivers’ capacity (43). As the aging population increases, the risk of institutionalization increases, particularly for those experiencing physical, cognitive, or social decline (44). Although institutionalization can provide necessary round-the-clock care, it often comes with psychological costs, including loss of autonomy and familiar surroundings (45).

#### 2.1.7 Fractures and falls

In the population over 65 years of age, fractures are the main manifestation of osteoporosis. Osteoporosis does not directly cause disability or death, but increases the risk of fractures from low-energy trauma. Femoral neck fractures almost always require hospitalization and have long-term consequences such as pain, chronic disability, and premature death. Femoral fractures have significant social costs: immediate post-fracture mortality is 5%, rising to 20% within a year, 30% of patients suffer permanent disability, 40% lose independent mobility, and 80% cannot perform at least one daily living activity independently (46). Several authors report both fractures and falls as frailty-related outcomes, and this is because these two outcomes are strictly related and possibly one is the consequence of the other.

#### 2.1.8 Emergency room access

Emergency room (ER) access, particularly for urgent cases (high priority), is a critical outcome in older adults (47). These visits often result from falls, exacerbation of chronic diseases, or sudden onset of severe symptoms (48). Emergency room access in high priority (ER-HP) cases among older adults typically involve life-threatening conditions such as stroke, heart attack, or severe trauma (49). However, even non-high-priority ER visits in frail older adults can indicate declining health and increased vulnerability (50). Furthermore, the period immediately after discharge from the ER is particularly critical, with a high risk of medication errors, falls, and exacerbation of chronic conditions (51). Finally, stress and disorientation associated with an emergency department visit can lead to delirium or cognitive decline in some frail older adults, underscoring the need for careful post-ER monitoring and support (52).

#### 2.1.9 Dementia

Dementia is a neurocognitive disorder characterized by impairments in memory, language, and other cognitive functions that affect an individual’s ability to perform daily activities. It is one of the leading causes of disability and dependency among the old age population (53, 54). According to the WHO Global Action Plan 2017–2025, dementia affected 47 million people worldwide in 2015, projected to increase to 75 million by 2030 and 132 million by 2050, with about 10 million new cases annually (one every 3 s). Costs exceed a trillion dollars annually, posing ongoing challenges for healthcare services. In 2019, the WHO estimated that more than 48 million people 65 years and older lived with dementia, representing 6.9% of the global old age population (55).

### 2.2 Data

When the objective is to identify frail individuals across the population and stratify them within a public health framework, administrative health data are needed.

In Italy, the implementation of health care delivery follows the institutional architecture of the Italian National Health System, with the Regional Authorities taking responsibility for their organization and planning. Italy is administratively divided into 20 regions, whose governments have the role of fulfilling the objectives of the National Health Plan at the regional level. They also coordinate and control local health units (LHUs), which are geographically distributed public organizations that provide public health and community health services, primary care and hospital care. Healthcare data are available for management purposes in all Italian local health units and cover all assisted individuals, making this information low-cost and complete for the entire population. Administrative healthcare data consists of large databases containing sensitive information, so addressing privacy issues is a prerequisite for their use.

Thanks to a formal agreement with LHU 6 “Euganea,” which serves residents of the province of Padua, in north-east Italy, we have access to several anonymized administrative healthcare databases. We identified the study population from the Regional Health Registry consisting of 224,512 residents in the 100 municipalities of the province of Padua, under the jurisdiction of LHU 6, during 2018 and 2019 aged 65 years or older. Only individuals who relocated outside of the province during the two-year observation period were excluded. Thanks to the use of administrative healthcare data, we were able to observe the entire population of registered healthcare recipients based on the healthcare registry without missing data. This approach ensures that we include all individuals in our study, even those who did not experience any healthcare events during the observation period. In other words, our dataset comprehensively covers the entire registered population, not just those who actively interacted with the healthcare system. Eight administrative healthcare data sources were selected: the regional health registry, which also includes the death registry, necessary to identify the reference population; hospital discharge records, containing information on the type and duration of hospital admissions and up to six diagnoses; ER admissions, with information on the type of service required and diagnoses; territorial psychiatry, with information on the type of service required and diagnoses; integrated home care, with information on the number and duration of interventions provided to assisted individuals; ticket exemptions, with information on the pathology or economic situation benefiting from the exemption; territorial pharmaceuticals, with information on prescribed drugs; and non-hospital services for non-self-sufficient people, which contains information on care services that are provided outside the hospital environment, such as residential, home care, or other forms of long-term care. These sources collect different types of events that occurred in the analyzed population. Information from all sources under analysis was coded, integrated and associated with the populations studied.

### 2.3 Health outcomes

Our aim was to include all outcomes most associated with frailty according to the literature. However, due to the limitations of administrative data, we were only able to reliably reconstruct a subset of these outcomes. Based on the literature review and the available data, we have chosen the following ten adverse health outcomes in the population analyzed during 2018 and 2019: death, urgent unplanned hospitalization, access to the ER with high priority, hospitalization, avoidable hospitalization, repeated hospitalization, femur fracture, dementia, disability and institutionalization. These outcomes were not only among the most frequently cited in the literature but were also feasible to operationalize using only administrative healthcare data sources. Among the most frequently cited outcomes, we excluded “falls” because it was not possible to distinguish which adverse events recorded in healthcare data were connected to falls, and not all falls are recorded in administrative health data. However, fractures, which are considered in the set of ten outcomes, can be considered a good proxy for severe falls.

A frequently cited outcome we did not considered is the “length of hospital stay.” This is due to several factors: difficulty of computing administrative data, as admission and discharge dates are often withheld for privacy reasons; the varied reasons for extended stays, such as death or long-term institutionalization; and the complexity of defining this outcome in cases of repeated hospitalizations.

Other less commonly cited outcomes, such as “functional decline,” “mobility impairment,” and “life satisfaction and quality of life,” are not available in health administrative data, they can only be collected through surveys or more qualitative evaluations in social services data.

The operationalization of outcomes with administrative healthcare databases must consider both practical aspects related to data availability and conceptual ones related to the meaning of events in the framework of the definition of frailty. Table 1 describes all the specific choices we faced in the definition of outcomes. Some outcomes, such as disability and dementia, are the result of the union of information coming from several databases to find all individuals interested by each condition.

**Table 1:** Outcomes operationalization according to administrative healthcare dataflows, codes, and description.

Outcome	Administrative healthcare source	Codes	Description
Death	Regional health registry^a^, which includes the death registry		Subject died in 2018 or 2019.
Access to ER with high priority	Emergency room admissions	The individual has received at least one access to the emergency room with triage code “4”.^b^	Subject had at least one access to the ER with HP in 2018 and/or 2019.
Dementia	Ticket exemptions	People with ticket exemption records related to dementia (the Italian exemption code is 011).^c^	Subject has a diagnosis of dementia in 2018 and/or 2019.
	Hospital discharge records, emergency room admissions	ICD-9-CM: 290, 331.2	
	Territorial psychiatry	ICD-10: F01 – F03, F05	
	Territorial pharmaceuticals	Use of anti-dementia drugs (ATC code: N06D)	
Disability	Ticket exemptions	People with ticket exemption records related to disability^b^^,^^d^: 3C1, 3C2, 3G1, 3G2, 3 L1, 3 L2, 3 L3, 3 M1, 3 M2, 3 M3, INAIL, G01, G02, L01, L02, L03, L04, S01, S02, S03, C01, C02, C03, C04	Subject is disabled in 2018 and/or 2019.
	Integrated home care	The individual has received at least one integrated home care intervention.	
Hospitalization	Hospital discharge records	The individual has received at least 1 day of hospitalization.	Subject had at least one hospitalization in 2018 and/or 2019.
Avoidable hospitalization	Hospital discharge records	The individual has received at least one admission with principal diagnosis (40): 1. Acute events: nutritional deficiencies, dehydration, bacterial pneumonia, ulcer bleeding, appendicitis with complications, urinary tract infections, pelvic inflammation. 2. Chronic events: diabetes, lower limb amputation in diabetic patients, hypertension, angina pectoris, heart failure, asthma.	Subject had at least one avoidable hospitalization in 2018 and/or 2019.
Emergency hospitalization	Hospital discharge records	The individual has received at least one admission that has the variable “type of admission” = 2 (urgent) or the variable “origin” = 2 (emergency room).	Subject had at least one emergency hospitalization in 2018 and/or 2019.
Repeated hospitalization	Hospital discharge records	The individual has received at least two hospitalizations during the same calendar year.	Subject had at least two hospitalizations in 2018 and/or 2019.
Femur fracture	Hospital discharge records, emergency room admissions	ICD-9-CM: 820	Subject had at least one femoral neck fracture in 2018 and/or 2019.
Institutionalization	Regional information flow for non-hospital services for non-self-sufficient older people	The individual has received at least 1 day of hospitalization in a residential care and/or nursing facility.	The subject was institutionalized for at least 1 day in the period between 2018 and 2019.

ER, emergency room; HP, high priority; ICD-9-CM, International Classification of Diseases, 9th Revision, Clinical Modification (https://archive.cdc.gov/www_cdc_gov/nchs/icd/icd9cm.htm); ICD-10, International Statistical Classification of Diseases and Related Health Problems, 10th Revision (https://www.cdc.gov/nchs/icd/icd-10/index.html); ATC, anatomical therapeutic chemical classification system (https://www.who.int/tools/atc-ddd-toolkit).

a This source contains also all assisted people in the Local Health Unit.

b This code is part of the triage system, which is standardized throughout Italy, following national guidelines. Code “4” is the most urgent case of triage codes on a scale from 1 to 4.

c The Italian exemption codes originate from the Italian National Health System. They are established by the Ministry of Health (https://www.salute.gov.it/BancheDati/anagrafi/MCR) and are used to identify different categories of exemption from healthcare co-payments. These codes are an integral part of the healthcare service management system in Italy.

d Ticket exemption records related to disability are: “3C1,” civilians with 100% disability; “C01,” civilians with 100% disability without attendance allowance; “C02,” civilians with 100% disability with attendance allowance; “3C2” or “C03,” civilians with a disability degree ranging from 67 to 99%; “C04,” civilians under 18 years of age with civil disabilities and attendance allowance; “3G1” or “G01,” war invalids from 1st to 5th category (https://www.anvcg.it/2025/06/24/tabelle-di-classificazione-delle-pensioni-di-guerra/); “3G2” or “G02,” war invalids from 6th to 8th category; “3 L1” or “L01,” severely work-disabled individuals with a disability degree from 80 to 100%; “3 L2” or L02, work-disabled individuals with a disability degree from 67 to 79%; “3 L3” or “L03,” work-disabled individuals with a disability degree from 1 to 66%; “INAIL” or “L04,” people injured at work or affected by occupational diseases are granted a temporary exemption valid only for the duration of the injury or illness; “3 M1” or “S01,” service-disabled individuals of 1st category (from 80 to 100%, https://www.osservatorioamianto.it/causa-servizio-requisiti-benefici/); “3 M2” or “S02,” service-disabled individuals from 2nd to 5th category (from 50 to 79%); “3 M3” or “S03,” service-disabled individuals from 6th to 8th category (from 20 to 49%).

### 2.4 Statistical analysis

Crude and standardized prevalences by sex and age were calculated for each of the ten frailty outcomes considered. The prevalences were estimated separately for 2018 and 2019. For example, in the 2018 cohort, the prevalence was calculated as the ratio of the number of events during the year to the average population, calculated as the mean number of individuals present at the beginning and at the end of the year. Standardized prevalences were calculated for men and women, using the direct standardization technique, with the average population of the 2 years as reference.

To measure and observe correlation among outcomes, we computed Kendall’s Tau for all combinations of outcomes. We then proceeded with a factor analysis of the outcomes, with the aim of verifying the structure of the relationships between the outcomes and capturing the presence of underlying patterns. Starting from the initial solution deriving from the principal component analysis, we considered both a factor analysis with orthogonal rotated factors and with correlated factors (promax rotation). The criterion used to select the number of factors was the number of eigenvalues greater than one from the variance–covariance matrix. Factor analysis was also performed by sex and age to assess whether different patterns of factors emerged, specifically for males aged 65–74, females aged 65–74, males aged 75 and over, and females aged 75 and older.

As the final step of our analysis, we estimated the conditional dependence associations between the variables using graphical models. This multivariate analysis approach aims to estimate and represent the network structure that describes the relationships between variables. We estimated an Ising model to represent the conditional structure through pairwise associations (56). This model is equivalent to a log-linear model that incorporates the main effects of the variables and their first order (pairwise) interactions. This approach quantifies the strength of the conditional association between two variables, i.e., pair associations net of other variables, with a conditional odds ratio (OR), which also indicates whether an edge is present on the graph or not. The estimation process occurred in two phases (57): first, we estimated the structure and then, as a second step, we estimated the model parameters. To learn the graphical structure, we estimated node-wise logistic regressions for each node, using stepwise variable selection based on the extended Bayesian Information Criteria (EBIC) score (56, 58, 59). Subsequently, we estimated a log-linear model including the main effects and the pairwise (first order) interactions found in the first step. The model was estimated by also accounting for sex and age (dichotomous: 65–74 and 75+).

## 3 Results

### 3.1 Health outcome prevalences

Crude and standardized prevalences (as percentages) are represented for the ten frailty outcomes analyzed, separately for the years 2018 (Supplementary Table 2) and 2019 (Table 2). The data for the 2 years are very consistent. The prevalence of outcomes is highly variable: the least common outcome is femur fracture, with a prevalence of 0.63% (95% CI: 0.59–0.66) in 2019. The most common outcome is disability, with a prevalence of 26.92% (95% CI: 26.73–27.11) in 2019.

**Table 2:** Crude and standardized prevalences (%) of the 10 outcomes observed in 2019.

Crude and standardized prevalences (%)						
Outcome 2019	Crude prevalences			Standardized prevalences		
	Female	Male	Whole population	Female	Male	Ratio F/M^a^
Death	3.46	3.87	3.64	2.77	4.25	0.65
ER access with highest priority	0.97	1.10	1.03	0.79	1.18	0.67
Femur fracture	0.83	0.37	0.63	0.70	0.40	1.75
Hospitalization	15.86	19.41	17.41	14.99	19.71	0.76
Repeated hospitalization	5.00	6.67	5.73	4.68	6.78	0.69
Emergency hospitalization	14.01	17.20	15.40	13.06	17.53	0.75
Avoidable hospitalization	4.63	5.50	5.01	3.95	5.80	0.68
Disability	28.13	25.36	26.92	24.67	26.35	0.94
Dementia	3.94	2.91	3.49	3.30	3.12	1.06
Institutionalization	1.32	0.85	1.11	1.09	0.90	1.21

ER, emergency room.

a Ratio between the female and male standardized prevalences.

Hospitalization and its three special cases (repeated hospitalizations, urgent hospitalizations, and avoidable hospitalizations) show higher prevalence in men than in women, with the gap widening after age standardization. Similar patterns are observed for mortality and emergency room access with the highest priority. On the contrary, femur fracture, dementia, and institutionalization are more common in women. Regarding disability, the crude prevalence appears to be higher in women, but after age standardization, the prevalence is higher among men. This suggests that the initially higher prevalence in women is due to their older age distribution, as women generally live longer. As observed in this paragraph, there are common outcomes, such as hospitalization, and rare ones, such as femur fracture; all are important as they represent different aspects of frailty, regardless of their frequency.

### 3.2 Emerging patterns from correlation and factor analysis

**Figure 3:**
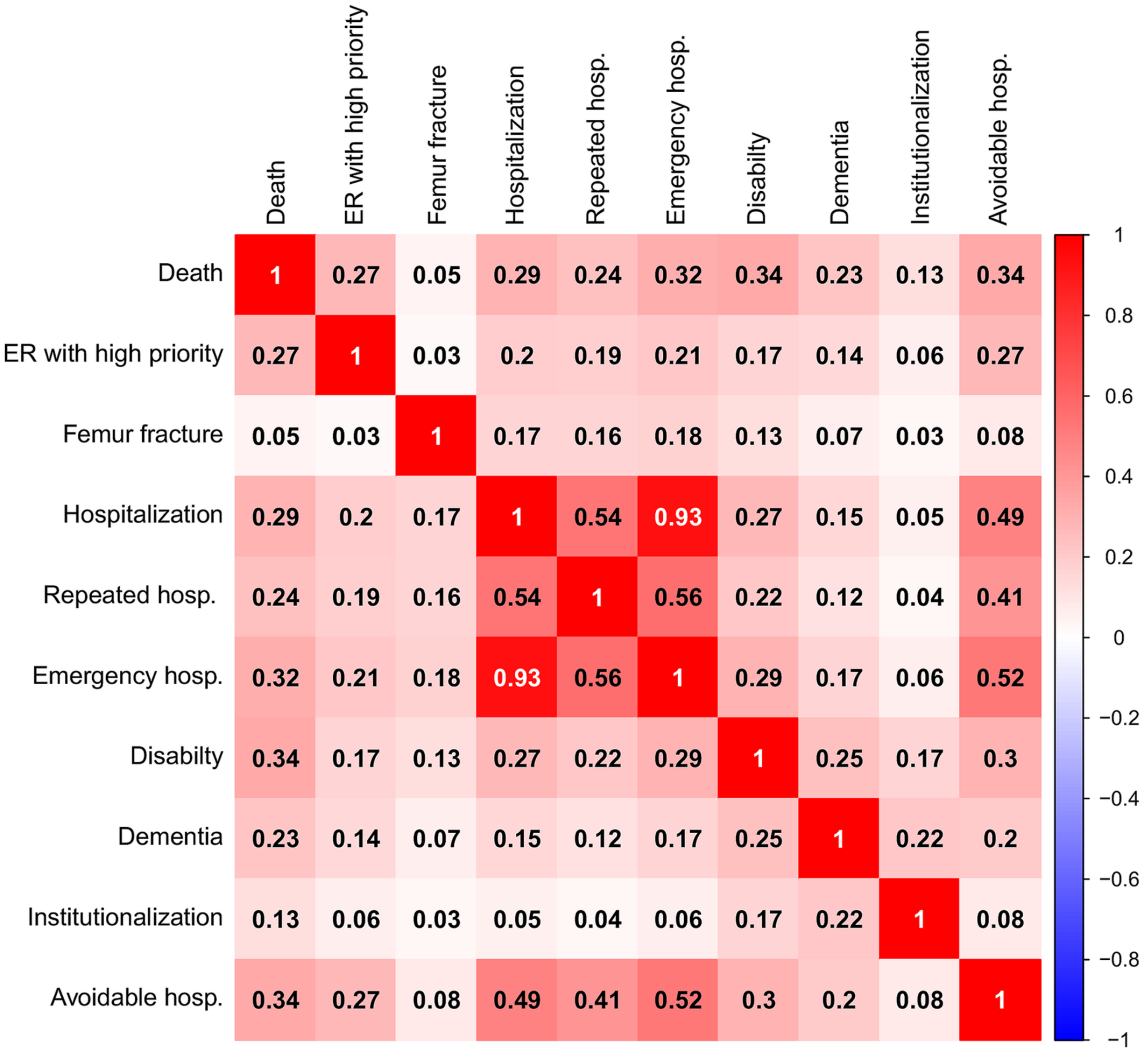
Kendall’s Tau correlation matrix of the 10 outcomes (all *p*-values are <0.0001).

The analysis of the Kendall correlation matrix (Figure 3) suggests the presence of factor patterns, as evidenced by the high correlations between some variables and the low correlations between others. There is an extremely high correlation between hospitalization and urgent admission (0.93), between hospitalization and avoidable admission (0.49), and between hospitalization and repeated hospitalization (0.54). This is expected since 90% of admissions are urgent, 30% are avoidable, and 36% are repeated. Furthermore, 88% of individuals who entered the ER with high priority also required emergency admission. The latter two outcomes overlap significantly in meaning.

The factor analysis identified three factors, with the third factor being less important than the first two. The factors are intercorrelated, particularly the first and second, which showed a correlation of 0.35. This suggests that factor solutions should be considered after an oblique rotation of the axes (promax). Following this rotation, the three factors together explained 64% of the total variance (32.3% for the first factor, 20% for the second and 11.9% by the third). The interpretation of the three factors is clearly evident in Table 3:

- The first factor is characterized by hospitalization events (hospital admission, urgent admission, repeated hospitalization, and avoidable admission), i.e., critical events resulting from compromised health conditions.
- The second factor is characterized by conditions of vulnerability and impaired personal autonomy: dementia, disability, and institutionalization.
- The third factor is essentially driven by a single variable: femur fracture. The proportion of explained variance is only slightly higher than the average variance explained by a single variable, indicating that it cannot be considered as a true factor. Nonetheless, this also highlights the importance of considering this variable independently.

**Table 3:** Factor patterns (promax rotation).

Outcome	Rotated factor pattern		
	Factor 1	Factor 2	Factor 3
Death	0.245	0.387	−0.385
Access to ER-HP	0.196	0.146	−0.570
Femur Fracture	0.285	0.261	0.731
Hospitalization	0.940	−0.087	0.057
Repeated hospitalization	0.770	−0.071	0.053
Emergency hospitalization	0.949	−0.064	0.048
Disability	0.196	0.554	−0.038
Dementia	−0.060	0.708	−0.033
Institutionalization	−0.222	0.745	0.135
Avoidable hospitalization	0.618	0.104	−0.235

ER-HP, emergency room access with highest priority.

The analysis conducted separately by sex and age groups (see the Supplementary Tables 3–6) confirmed the presence of the first two factors, while the third factor was sometimes replaced by the variables of death and access to the ER access with high priority, particularly in the 65–74 age group. This factor is likely driven by cardiovascular events, many of which result in death. In summary, factor analysis distinguishes critical events from chronic conditions. Note that death, the final stage of the other outcomes, does not characterize first or second factor, as it is a possible consequence of both the first and the second. In conclusion, the outcomes of hospitalization, emergency hospitalization, repeated hospitalization and avoidable hospitalization can be attributed to the same factor that represents critical events. Avoidable hospitalization is an outcome more closely related to the management of healthcare resources than to an individual’s health status, making it less relevant for assessing frailty. This is also supported by the limited references in the literature that discuss it (Figure 2).

Considering all these outcomes simultaneously is not meaningful when adopting a predictive approach to constructing a frailty index, as this would risk overrepresenting the predictive variables associated with such outcomes. A selection must therefore be made, and it appears appropriate to focus on a single event, preferably one with the highest factor loadings, namely, hospitalization and urgent admission. Our final choice falls on hospitalization, as it encompasses both urgent admission and the other types of hospital admissions considered in the analysis. Moreover, it is a variable that is straightforward to construct and can be compared across different information systems, unlike urgent admission, which lacks standardized coding across regional information systems. In the following analysis, we will only consider general hospitalization and exclude the other three variables that describe specific cases of hospitalization. All the other variables will be retained, since neither of them is a subset of another.

### 3.3 Visualizing outcomes connections

**Figure 4:**
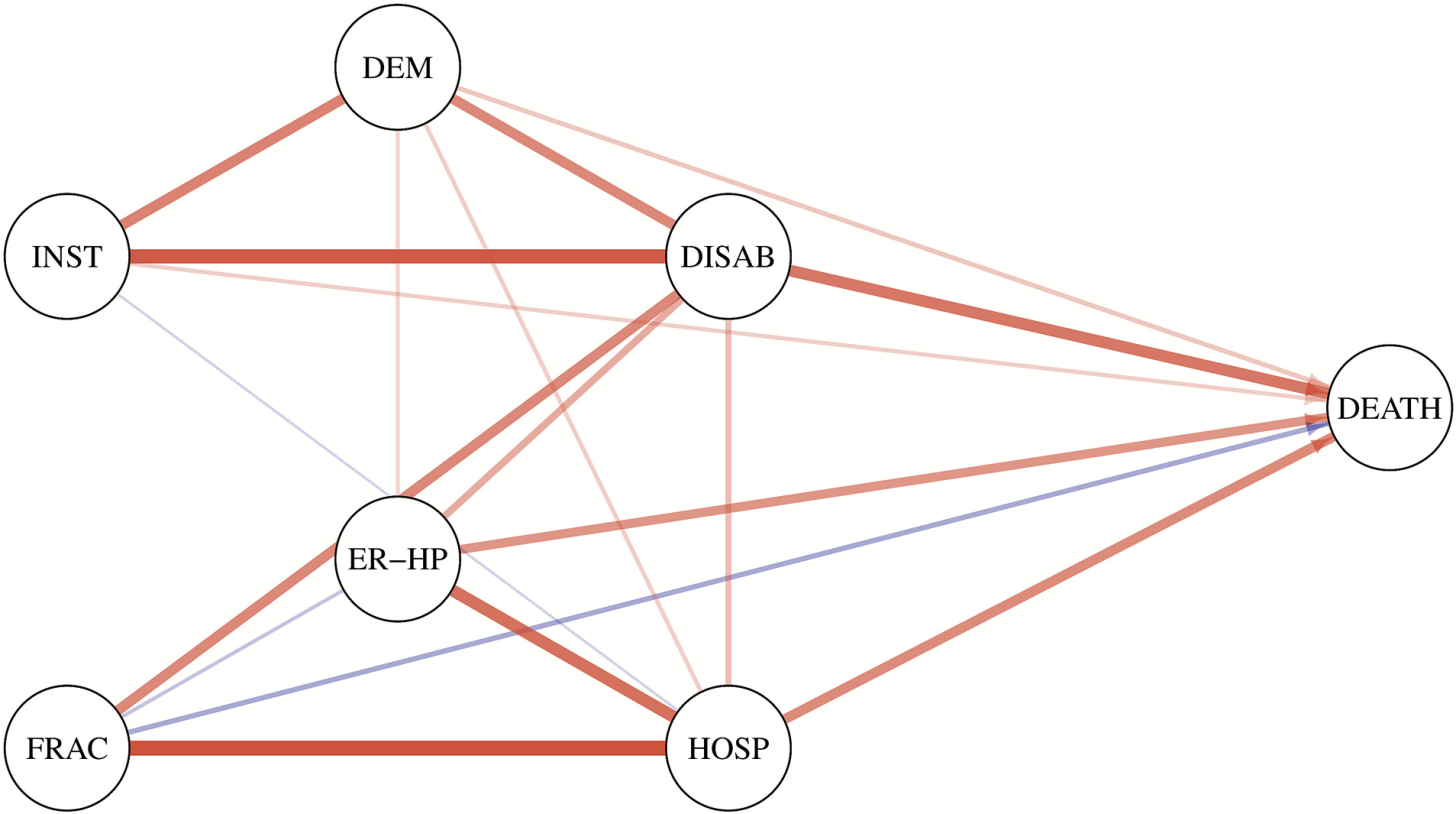
Estimated network with the selected adverse health outcomes. Conditional associations are represented by edges, whereas conditional independence between two variables is indicated by the absence of a connecting edge. The thickness and color intensity of each edge are proportional to Yule’s Q coefficients, which are computed from the estimated conditional odds ratios shown in the lower triangular of the matrix in Figure 5. Red edges indicate positive associations, whereas blue edges represent negative associations. DEM, dementia; DISAB, disability; INST, institutionalization; ER-HP, ER with high priority; FRAC, femur fracture; HOSP, hospitalization.

**Figure 5:**
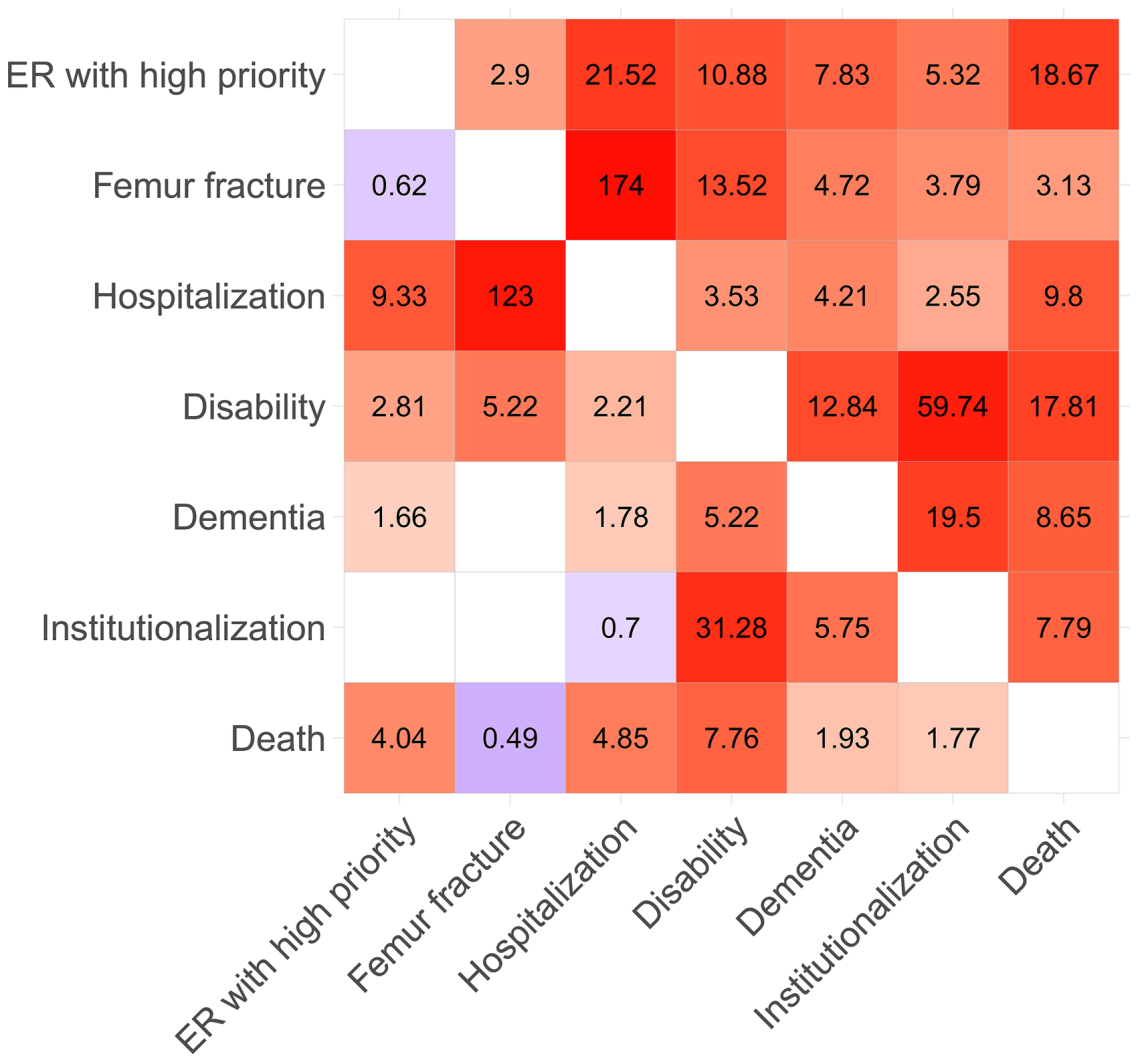
Matrix with the estimated marginal (upper triangular) and conditional (lower triangular) odds ratio. The color intensity of the cells is proportional to Yule’s Q coefficients, computed from the odds ratio associated with each cell. Red cells indicate positive associations, whereas blue cells represent negative ones, while blank cells indicate conditional independence between the corresponding variables. Yule’s Q coefficient is given by (OR-1)/(OR+1), the index stands between −1 and 1.

Figure 4 shows the estimated network of the selected adverse health outcomes. The circles (nodes) represent the variables, while the lines (edges) indicate the presence of conditional associations between them. Each edge represents a conditional association between two connected variables, which is the net pairwise relationship after controlling for the influence of the other variables and is expressed as a conditional OR. A line between two variables means that they are conditionally dependent, given all the other variables in the graph. Conversely, if two nodes are not connected by an edge, then the two variables are conditionally independent, given the effect of the other variables. Edges are undirected between all variables except for death. Since the other conditions are measured at the same time point, it is not possible to establish a clear chronological order among them. On the contrary, death is considered the final outcome, occurring chronologically after all other conditions, so the edges are directed from the other variables toward death. The thickness of the lines reflects the strength of the associations, while the colors represent the direction of the effect: in red, the positive associations, while in blue the negative ones. The thickness and intensity of the color of the edges are proportional to the Yule’s Q coefficient computed using the conditional OR estimated by the model and reported in the lower triangular part of the matrix in Figure 5.

The upper triangular part of the matrix in Figure 5 shows the pairwise marginal OR calculated for the outcomes considered, while the lower triangular part shows the conditional OR estimated by the model. Empty cells indicate that the two variables are conditionally independent and correspond to a missing edge in the graph.

Disability plays a central role, being strongly and significantly related to all other outcomes. Another outcome that appears to play a relevant role is hospitalization. Together, these two outcomes are the most important nodes in the graph: the former represents a severe condition of psychophysical decline, while the latter signifies a general critical event, as indicated by the two factors in the principal component analysis. Moreover, their conditional association is not particularly strong when considering the effect of the other outcomes in the graph.

The three outcomes most strongly associated with death are hospitalization, access to the emergency room with high priority, and disability. Although the marginal odds ratio between dementia and death is particularly high, the same relationship becomes less intense when considering the conditional OR, which accounts for the effect of the other variables. This result suggests that it is not dementia that is directly related to death, but rather its association with other variables that are more strongly related to it, such as disability. Death is positively associated with all outcomes, except fractures. A possible explanation could be that old people can rapidly decline after a fracture, leading to mobility problems (disability) rather than directly to death.

In Figure 4, we can observe the existence of a relationship between access to the ER with high priority and hospitalization, which are two conceptually connected events. Indeed, after very severe and urgent access to the ER, an older person may need to be monitored and probably treated during hospitalization. The connection between femur fracture and hospitalization follows the same logic; indeed, 98.5% of all femur fractures in the observed population were hospitalized. In fact, most fractures need to be treated, and especially if they are the result of a fall, patients need to be monitored for further consequences.

Figure 4 highlights also a strong relationship between disability, dementia, and institutionalization. This suggests that these three variables are highly associated, reflecting a close relationship between being disabled, having dementia, and requiring institutionalization, as a graphical confirmation of the presence of the second factor underlined by the factor analysis described in paragraph 3.2. The connection might also follow a consequential pattern that leads older people with severe dementia or disability to need institutional care. Regarding institutionalization, the conditional OR linking institutionalization to death is weaker compared to the marginal one. This suggests that it is not institutionalization itself that leads to death, but rather its association with other outcomes. Furthermore, it is interesting to note that although institutionalization shows a strong conditional association with dementia and disability, its relationships with other outcomes remain mild.

Institutionalization is a rare outcome that appears to be a logical consequence (though not strictly causal) of dementia and disability and is only mildly associated with the other outcomes. Furthermore, institutionalization may not directly reflect the health condition of the older person but rather a decision made by the close family members, who may or may not have the resources to manage care at home. Thus, this decision is influenced by various factors beyond the individual’s health status. Therefore, institutionalization is excluded from the fundamental and parsimonious set of representative outcomes related to frailty.

As a broader interpretation, we were also able to identify the most relevant predictors for death among the other outcomes, which can be distinguished into groups according to their impact. The presence of disability emerged as the most useful predictor, with a conditional odds ratio (OR) that is almost double that of high-priority emergency room (ER) access and hospitalization. Indeed, these events increase the level of stress, reduce the independence of an older individual, and worsen their overall health condition in a way that may not be recoverable, potentially escalating into death.

Outcomes that have a mild impact on death include dementia and institutionalization, as they indicate poor general conditions both in terms of health and autonomy. However, after accounting for the other outcomes, they have a limited impact on their own. Interestingly, femur fracture seems to have a mild protective effect on mortality. After accounting for the other outcomes, we may hypothesize that older individuals who only experience femur fractures in the absence of other negative health conditions may have sustained the injury because of a more active lifestyle and subsequently recover their previous health status without progressing to death.

In conclusion, our analyzes have led us to identify a set of six outcomes that effectively highlight various facets of frailty without being redundant. These outcomes collectively capture frailty multidimensional nature while avoiding unnecessary duplication. They are death, high-priority Emergency Room access, femur fracture, hospitalization, disability and dementia.

## 4 Discussion

Measuring health-related frailty in the population has become a strategic objective for the Italian National Health Service. Considering the aging of the population and the simultaneous reduction in available resources, it is essential to adopt targeted care strategies and to identify those segments of the population most at risk of adverse health outcomes. Stratifying the population according to the levels of frailty and the corresponding care needs is increasingly emphasized in national guidelines and reiterated in recent regulations aimed at reforming healthcare delivery through the resources of the National Recovery and Resilience Plan (PNRR) (60).

The operational approach widely adopted in the literature within this context is a predictive one. This relies on the development of frailty indices using variables that best predict adverse health outcomes such as death, hospitalization, disability, and others (61–63). Typically, the frailty index is a linear combination of such variables obtained through regression models, or it may be the sum of such variables, or derived through more sophisticated statistical approaches, necessary to extrapolate the subset of variables that best predict multiple health outcomes simultaneously [e.g., (14, 16, 37, 64)]. However, what is not addressed in the literature is which and how many health outcomes should be predicted, as some studies focus solely on death, others on hospitalization, some consider a couple of outcomes like hospitalization and disability, and so on. It is clear that, depending on the outcomes considered, the set of predictors will change, thus altering the composition of the frailty index. Moreover, focusing on a single outcome is insufficient, as frailty is a condition of general vulnerability that may manifest with multiple outcomes.

Therefore, our study aimed to answer the following question: which and how many adverse health outcomes should be considered when constructing a frailty index for the population using a predictive approach?

Our analysis led us to consider the following adverse health outcomes:

1. Death: the ultimate and most severe outcome, strongly associated with hospitalization, high-priority emergency room access, and disability.
2. High-priority ER access: representing a critical event that often leads to hospitalization and is associated with increased risk of mortality.
3. Femur fracture: a major event among older adults, frequently requiring hospitalization and potentially resulting in disability.
4. Hospitalization: a moderately critical event that includes various types of hospital admissions.
5. Disability: a central outcome that reflects substantial deterioration, strongly linked to dementia and an increased risk of mortality.
6. Dementia: a chronic condition associated with disability.

For constructing frailty indices using the predictive approach, we therefore propose considering these six adverse health outcomes, which include both adverse events (high-priority ER access and hospitalization), and adverse conditions (disability and dementia), and death. We believe that this work is an important starting point for developing frailty indices using a predictive approach, as it provides a robust set of health outcomes from which to identify the set of variables predictive of these outcomes, i.e., the set of variables that make up the frailty index.

It is important to emphasize that in this work, we refer to the construction of frailty indices aimed at stratifying the entire population of a territory, identified via the health registry. The adverse health outcomes discussed are those identifiable in administrative health data and not others, such as functional decline or mobility impairment. However, as shown in Figure 2, most of the adverse outcomes considered in the literature can be identified through administrative health data. We are referring to health outcomes that are already quite severe, and are therefore inevitably captured through healthcare flows. Administrative data do not capture those health outcomes that may be consequences of frailty or pre-frailty and do not lead to official records. For example, the inability to perform certain activities of daily living, which has not yet been certified through exemptions or other forms of recorded protection.

The strength of administrative health data lies in its ability to include the entire resident population and stratify the population, including healthy individuals, thus enabling targeted actions for the most frail individuals. A significant limitation of administrative data is the absence of social, economic, and lifestyle information, which are crucial factors for explaining health outcomes.

Regarding the predictive approach used in this study, it should be noted that this is an operational approach to the construction of frailty indices, widely used, but there are theoretical approaches in the literature that provide frailty indicators not based on predictive capacity. These are well-known and widely used, but they cannot be replicated with administrative health data. The most well-known example is the Fried phenotypic frailty index (35), based on five items (unintentional weight loss, exhaustion, low physical activity, slow walking speed, and weakness), which cannot be identified from administrative health data.

Finally, it should be noted that our analysis was based on the older adults of the province of Padua (North-East Italy). Although the analysis is based on a large population (over 200,000 residents), comparison with other regions might reveal differences in the relationships between outcomes. These potential differences could arise due to variations in the composition of the older population across regions or differences in coding practices for the analyzed health outcomes in different parts of the country.

## 5 Conclusion

This study aimed to identify which and how many adverse health outcomes should be considered when constructing a frailty index using a predictive approach based on administrative health data. Through factor analysis and graphical modeling, we identified six key outcomes: death, high priority emergency room access, femur fracture, hospitalization, disability, and dementia, as central and nonredundant variables. Our findings provide a methodologically grounded selection of outcomes that are both clinically meaningful and feasible to extract from routinely available data, offering a solid foundation for developing population-based frailty indices.

## Data Availability

The datasets presented in this article are not readily available because the data supporting this study’s findings are held by the 6 LHU-Veneto region and were used under license for this work, but they are not available to the general public. Requests to access the datasets should be directed to GB, giovanna.boccuzzo@unipd.it.

## Footnotes

1 Given that the purpose of our literature review differs substantially from those typically associated with the PRISMA 2020 Checklist, only a subset of the checklist items were applicable to our study.
