# Supplementary material for: Identifying core adverse health outcomes for frailty assessment in older adults using administrative data
Source: Front Med (Lausanne). 2025 Dec 15;12:1678317. doi: 10.3389/fmed.2025.1678317 (PMC12745202; doi:10.3389/fmed.2025.1678317)
Supplement: Supplementary file 1 [file Data_Sheet_1.PDF]

Supplementary Material

1 Supplementary Figures and Tables

1.1 Literature review

Outcome	Number of citations	Articles
Death[1]	95	Abraham (2021), Andrew (2008), Armstrong (2015), At (2015), Avila-Funes (2009), Bennett (2013), Bilotta (2012), Blodgett (2021), Buchman (2011), Cao (2022), Cavazza (2014), Chen (2020), Chen (2023), Chin A Paw (1999), Choe (2018), Clegg (2016), Daniels (2012), De la Garza Ramos (2016), Drubbel (2013), Ensrud (2007), Ensrud (2008), Ensrud (2009), Falasca (2011), Forti (2012), Forti (2014), Fried (2001), Garcìa-Garcìa (2014), Gilbert (2018), Gill (2010), Gobbens (2014), Graham (2009), Gu (2009), Gu (2016), Hall (2017), Hall (2022), Hanlon (2023), Hastings (2008), Hogan (2012), Jauhari (2020), Jones (2004), Jones (2005), Joosten (2014), Kamaruzzaman (2010), Khandelwal (2012), Kim (2018), Kim (2019), Klein (2005), Kulminski (2008), Kundi (2021), Le Pogam (2022), Liang (2021), Lucicesare (2010), Ma (2009), Mazzaglia (2007), McIsaac (2019), Mitnitski (2002), Moldovan (2020), Morley (2012), Nishimura (2022), O'Caoimh (2014), Orkaby (2019), Pajewski (2019), Pandolfi (2016), Pilotto (2008), Quach (2022), Ravaglia (2008), Rockwood (1994), Rockwood (1999), Rockwood (2005), Rockwood (2005b), Rockwood (2006), Romero-Ortuno (2013), Romero-Ortuno (2014), Rothman (2008), Saliba (2001), Searle (2008), Segal (2017), Segal (2017b), Sharma (2022), Shashikumar (2020), Shen (2021), Shi (2011), Silan (2022), Sirola (2011), Song (2010), Soong (2015), Soong (2019), Tarekegn (2020), Tew (2021), Theou (2013), Velanovich (2013), Vetrano (2023), Woo (2012), Woods (2005), Zhao (2022)

[1] Different time intervals are considered in the articles.

Disability	27	Amici (2008), Avila-Funes (2009), Boyd (2005), Buchman (2011), Carrière (2005), Chin A Paw (1999), Daniels (2012), Ensrud (2008), Ensrud (2009), Forti (2012), Forti (2014), Fried (2001), Gobbens (2012), Kiely (2009), Kim (2018), Kim (2019), Kim (2020), Puts (2005), Ravaglia (2008), Romero-Ortuno (2014), Rothman (2008), Segal (2017), Silan (2022), Tarekegn (2020), Tom (2013), Woods (2005), Wu (2020)
Hospitalization	25	Abraham (2021), Avila-Funes (2009), Bilotta (2012), Choe (2018), Daniels (2012), Forti (2012), Forti (2014), Fried (2001), Garcìa-Garcìa (2014), Hall (2022), Hanlon (2023), Hastings (2008), Hogan (2012), Kamaruzzaman (2010), Kiely (2009), Kim (2019), O'Caoimh (2014), Pajewski (2019), Ravaglia (2008), Rockwood (1994), Segal (2017), Segal (2017b), Vetrano (2023), Woods (2005), Wu (2020)
Institutionalization	22	Brody (1997), Brody (2002), Clegg (2016), Drubbel (2013), Forti (2012), Hastings (2008), Hogan (2012), Jones (2004), Kamaruzzaman (2010), Kim (2019), Le Pogam (2022), McIsaac (2019), Moldovan (2020), O'Caoimh (2014), Rockwood (1994), Rockwood (1999), Rockwood (2005), Rockwood (2006), Rothman (2008), Segal (2017), Segal (2017b), Soong (2015)
Falls	14	Abraham (2021), Bilotta (2012), de Vries (2013), Ensrud (2007), Ensrud (2008), Ensrud (2009), Fried (2001), Kiely (2009), Kim (2018), Pajewski (2019), Rothman (2008), Samper-Ternent (2012), Segal (2017b), Tom (2013)
Fractures[2]	13	de Vries (2013), Ensrud (2007), Ensrud (2008), Ensrud (2009), Forti (2012), Forti (2014), Ravaglia (2008), Segal (2017), Segal (2017b), Silan (2022), Tarekegn (2020), Tom (2013), Woods (2005)
Length of hospital stay (LOS)	10	Gilbert (2018), Khandelwal (2012), Le Pogam (2022), Liang (2021), Makary (2010), Rockwood

[2] Some articles consider generic fractures, while others focus on fractures at specific points.

		(1994), Sharma (2022), Shashikumar (2020), Soong (2019), Zhao (2022)
Repeated hospitalization	9	Crane (2010), Gilbert (2018), Kundi (2021), Le Pogam (2022), Lekan (2017), Sharma (2022), Soong (2015), Soong (2019), Tew (2021),
Emergency room access[3]	7	Abraham (2021), Drubbel (2013), Hastings (2008), Kiely (2009), Pajewski (2019), Silan (2022), Tarekegn (2020)
Emergency admission	6	Cavazza (2014), Clegg (2016), Falasca (2011), Pandolfi (2016), Silan (2022), Tarekegn (2020)
Dementia	6	Avila-Funes (2009), Avila-Funes (2012), Buchman (2007), Gray (2013), Solfrizzi (2013), Song (2011)
Functional decline	5	Brown (2000), Chin A Paw (1999), Puts (2005), Saliba (2001), Walker (2005)
Mobility impairment[4]	5	At (2015), Kim (2018), Segal (2017b), Woo (2012), Wu (2020)
Avoidable hospitalization	4	Mazzaglia (2007), Silan (2022), Tarekegn (2020), Wu (2020)
Life satisfaction and quality of life	3	Gobbens (2012), St John (2013), Strawbridge (1998)
Post-operative complications	3	De la Garza Ramos (2016), Makary (2010), Velanovich (2013)
Use of health and community services	2	Gobbens (2012), Rochat (2010)
Long-term care[5]	2	Nishimura (2022), Rockwood (1994)
Cognitive impairment	2	Buchman (2011), Khandelwal (2012)

[3] General or high priority emergency room access.

[4] Including: dependency, physical limitation, help with IADL, mobility and self-care and housework.

[5] Including home assistance.

Lean mass index decline	1	Jung (2014)

Table 1: The 123 scientific articles considered in the literature review are listed by health outcomes.

References:

Abraham, D. S., Pham Nguyen, T. P., & Willis, A. W. (2021). Claims-Based Frailty and Outcomes: Applying an Aging Measure to Older Adults with Parkinson's Disease. *Movement disorders : official journal of the Movement Disorder Society*, *36*(8), 1871–1878. https://doi.org/10.1002/mds.28561

Amici, A., Baratta, A., Linguanti, A., Giudice, G., Servello, A., Scalise, C., Tafaro, L., Cicconetti, P., Marigliano, V., & Cacciafesta, M. (2008). The Marigliano-Cacciafesta polypathological scale: a tool for assessing fragility. *Archives of gerontology and geriatrics*, *46*(3), 327–334. https://doi.org/10.1016/j.archger.2007.05.007

Andrew, M. K., Mitnitski, A. B., & Rockwood, K. (2008). Social vulnerability, frailty and mortality in elderly people. *PloS one*, *3*(5), e2232. https://doi.org/10.1371/journal.pone.0002232

Armstrong, J. J., Mitnitski, A., Launer, L. J., White, L. R., & Rockwood, K. (2015). Frailty in the Honolulu-Asia Aging Study: deficit accumulation in a male cohort followed to 90% mortality. *The journals of gerontology. Series A, Biological sciences and medical sciences*, *70*(1), 125–131. https://doi.org/10.1093/gerona/glu089

At, J., Bryce, R., Prina, M., Acosta, D., Ferri, C. P., Guerra, M., Huang, Y., Rodriguez, J. J., Salas, A., Sosa, A. L., Williams, J. D., Dewey, M. E., Acosta, I., Liu, Z., Beard, J., & Prince, M. (2015). Frailty and the prediction of dependence and mortality in low- and middle-income countries: a 10/66 population-based cohort study. *BMC medicine*, *13*, 138. https://doi.org/10.1186/s12916-015-0378-4

Avila-Funes, J. A., Amieva, H., Barberger-Gateau, P., Le Goff, M., Raoux, N., Ritchie, K., Carrière, I., Tavernier, B., Tzourio, C., Gutiérrez-Robledo, L. M., & Dartigues, J. F. (2009). Cognitive impairment improves the predictive validity of the phenotype of frailty for adverse health outcomes: the three-city study. *Journal of the American Geriatrics Society*, *57*(3), 453–461. https://doi.org/10.1111/j.1532-5415.2008.02136.x

Avila-Funes, J. A., Carcaillon, L., Helmer, C., Carrière, I., Ritchie, K., Rouaud, O., Tzourio, C., Dartigues, J. F., & Amieva, H. (2012). Is frailty a prodromal stage of vascular dementia? Results from the Three-City Study. *Journal of the American Geriatrics Society*, *60*(9), 1708–1712. https://doi.org/10.1111/j.1532-5415.2012.04142.x

Bennett, S., Song, X., Mitnitski, A., & Rockwood, K. (2013). A limit to frailty in very old, community-dwelling people: a secondary analysis of the Chinese longitudinal health and longevity study. *Age and ageing*, *42*(3), 372–377. https://doi.org/10.1093/ageing/afs180

Bilotta, C., Nicolini, P., Casè, A., Pina, G., Rossi, S., & Vergani, C. (2012). Frailty syndrome diagnosed according to the Study of Osteoporotic Fractures (SOF) criteria and adverse health outcomes among community-dwelling older outpatients in Italy. A one-year prospective cohort

study. *Archives of gerontology and geriatrics*, *54*(2), e23–e28. https://doi.org/10.1016/j.archger.2011.06.037

Blodgett, J., Theou, O., Kirkland, S., Andreou, P., & Rockwood, K. (2015). Frailty in NHANES: Comparing the frailty index and phenotype. *Archives of gerontology and geriatrics*, *60*(3), 464–470. https://doi.org/10.1016/j.archger.2015.01.016

Boyd, C. M., Xue, Q. L., Simpson, C. F., Guralnik, J. M., & Fried, L. P. (2005). Frailty, hospitalization, and progression of disability in a cohort of disabled older women. *The American journal of medicine*, *118*(11), 1225–1231. https://doi.org/10.1016/j.amjmed.2005.01.062

Brody, K. K., Johnson, R. E., & Douglas Ried, L. (1997). Evaluation of a self-report screening instrument to predict frailty outcomes in aging populations. *The Gerontologist*, *37*(2), 182–191. https://doi.org/10.1093/geront/37.2.182

Brody, K. K., Johnson, R. E., Ried, L. D., Carder, P. C., & Perrin, N. (2002). A comparison of two methods for identifying frail Medicare-aged persons. *Journal of the American Geriatrics Society*, *50*(3), 562–569. https://doi.org/10.1046/j.1532-5415.2002.50127.x

Brown, M., Sinacore, D. R., Binder, E. F., & Kohrt, W. M. (2000). Physical and performance measures for the identification of mild to moderate frailty. *The journals of gerontology. Series A, Biological sciences and medical sciences*, *55*(6), M350–M355. https://doi.org/10.1093/gerona/55.6.m350

Buchman, A. S., Boyle, P. A., Wilson, R. S., Tang, Y., & Bennett, D. A. (2007). Frailty is associated with incident Alzheimer's disease and cognitive decline in the elderly. *Psychosomatic medicine*, *69*(5), 483–489. https://doi.org/10.1097/psy.0b013e318068de1d

Buchman, A. S., Leurgans, S. E., Boyle, P. A., Schneider, J. A., Arnold, S. E., & Bennett, D. A. (2011). Combinations of motor measures more strongly predict adverse health outcomes in old age: the rush memory and aging project, a community-based cohort study. *BMC medicine*, *9*, 42. https://doi.org/10.1186/1741-7015-9-42

Cao, X., Chen, C., Zhang, J., Xue, Q. L., Hoogendijk, E. O., Liu, X., Li, S., Wang, X., Zhu, Y., & Liu, Z. (2022). Aging metrics incorporating cognitive and physical function capture mortality risk: results from two prospective cohort studies. *BMC geriatrics*, *22*(1), 378. https://doi.org/10.1186/s12877-022-02913-y

Carrière, I., Colvez, A., Favier, F., Jeandel, C., Blain, H., & EPIDOS study group (2005). Hierarchical components of physical frailty predicted incidence of dependency in a cohort of elderly women. *Journal of clinical epidemiology*, *58*(11), 1180–1187. https://doi.org/10.1016/j.jclinepi.2005.02.018

Cavazza, G., & Malvi, C. (2014). La fragilità degli anziani. *Strategie, progetti, strumenti per invecchiare bene.* Maggioli Editore.

Chen, Q., Tang, B., Zhai, Y., Chen, Y., Jin, Z., Han, H., Gao, Y., Wu, C., Chen, T., & He, J. (2020). Dynamic statistical model for predicting the risk of death among older Chinese people, using

longitudinal repeated measures of the frailty index: a prospective cohort study. *Age and ageing*, *49*(6), 966–973. https://doi.org/10.1093/ageing/afaa056

Chen, X., Hou, C., Yao, L., Ma, Y., Li, Y., Li, J., Gui, M., Wang, M., Zhou, X., Lu, B., & Fu, D. (2023). The association between chronic heart failure and frailty index: A study based on the National Health and Nutrition Examination Survey from 1999 to 2018. *Frontiers in cardiovascular medicine*, *9*, 1057587. https://doi.org/10.3389/fcvm.2022.1057587

Chin A Paw, M. J., Dekker, J. M., Feskens, E. J., Schouten, E. G., & Kromhout, D. (1999). How to select a frail elderly population? A comparison of three working definitions. *Journal of clinical epidemiology*, 52(11), 1015–1021. https://doi.org/10.1016/s0895-4356(99)00077-3

Choe, Y. R., Joh, J. Y., Sunwoo, D., & Kim, Y. P. (2018). Interaction between frailty and nutritional status on mortality and long-term hospitalization in older Koreans: A retrospective analysis of data from the 2008 Survey on Health and Welfare Status of the Elderly in Korea. *Archives of gerontology and geriatrics*, *76*, 106–113. https://doi.org/10.1016/j.archger.2018.01.011

Clegg, A., Bates, C., Young, J., Ryan, R., Nichols, L., Ann Teale, E., Mohammed, M. A., Parry, J., & Marshall, T. (2016). Development and validation of an electronic frailty index using routine primary care electronic health record data. *Age and ageing*, *45*(3), 353–360. https://doi.org/10.1093/ageing/afw039

Crane, S. J., Tung, E. E., Hanson, G. J., Cha, S., Chaudhry, R., & Takahashi, P. Y. (2010). Use of an electronic administrative database to identify older community dwelling adults at high-risk for hospitalization or emergency department visits: the elders risk assessment index. *BMC health services research*, *10*, 338. https://doi.org/10.1186/1472-6963-10-338

Daniels, R., van Rossum, E., Beurskens, A., van den Heuvel, W., & de Witte, L. (2012). The predictive validity of three self-report screening instruments for identifying frail older people in the community. *BMC public health*, *12*, 69. https://doi.org/10.1186/1471-2458-12-69

De la Garza Ramos, R., Goodwin, C. R., Jain, A., Abu-Bonsrah, N., Fisher, C. G., Bettegowda, C., & Sciubba, D. M. (2016). Development of a Metastatic Spinal Tumor Frailty Index (MSTFI) Using a Nationwide Database and Its Association with Inpatient Morbidity, Mortality, and Length of Stay After Spine Surgery. *World neurosurgery*, *95*, 548–555.e4. https://doi.org/10.1016/j.wneu.2016.08.029

de Vries, O. J., Peeters, G. M., Lips, P., & Deeg, D. J. (2013). Does frailty predict increased risk of falls and fractures? A prospective population-based study. *Osteoporosis international : a journal established as result of cooperation between the European Foundation for Osteoporosis and the National Osteoporosis Foundation of the USA*, *24*(9), 2397–2403. https://doi.org/10.1007/s00198-013-2303-z

Drubbel, I., de Wit, N. J., Bleijenberg, N., Eijkemans, R. J., Schuurmans, M. J., & Numans, M. E. (2013). Prediction of adverse health outcomes in older people using a frailty index based on routine primary care data. *The journals of gerontology. Series A, Biological sciences and medical sciences*, *68*(3), 301–308. https://doi.org/10.1093/gerona/gls161

Ensrud, K. E., Ewing, S. K., Cawthon, P. M., Fink, H. A., Taylor, B. C., Cauley, J. A., Dam, T. T., Marshall, L. M., Orwoll, E. S., Cummings, S. R., & Osteoporotic Fractures in Men Research Group (2009). A comparison of frailty indexes for the prediction of falls, disability, fractures, and mortality in older men. *Journal of the American Geriatrics Society*, *57*(3), 492–498. https://doi.org/10.1111/j.1532-5415.2009.02137.x

Ensrud, K. E., Ewing, S. K., Taylor, B. C., Fink, H. A., Cawthon, P. M., Stone, K. L., Hillier, T. A., Cauley, J. A., Hochberg, M. C., Rodondi, N., Tracy, J. K., & Cummings, S. R. (2008). Comparison of 2 frailty indexes for prediction of falls, disability, fractures, and death in older women. *Archives of internal medicine*, *168*(4), 382–389. https://doi.org/10.1001/archinternmed.2007.113

Ensrud, K. E., Ewing, S. K., Taylor, B. C., Fink, H. A., Stone, K. L., Cauley, J. A., Tracy, J. K., Hochberg, M. C., Rodondi, N., Cawthon, P. M., & Study of Osteoporotic Fractures Research Group (2007). Frailty and risk of falls, fracture, and mortality in older women: the study of osteoporotic fractures. *The journals of gerontology. Series A, Biological sciences and medical sciences*, *62*(7), 744–751. https://doi.org/10.1093/gerona/62.7.744

Falasca, P., Berardo, A., & Di Tommaso, F. (2011). Development and validation of predictive MoSaiCo (Modello Statistico Combinato) on emergency admissions: can it also identify patients at high risk of frailty?. *Annali dell'Istituto superiore di sanita*, *47*(2), 220–228. https://doi.org/10.4415/ANN_11_02_15

Forti, P., Maioli, F., Lega, M. V., Montanari, L., Coraini, F., & Zoli, M. (2014). Combination of the clock drawing test with the physical phenotype of frailty for the prediction of mortality and other adverse outcomes in older community dwellers without dementia. *Gerontology*, *60*(3), 204–211. https://doi.org/10.1159/000356701

Forti, P., Rietti, E., Pisacane, N., Olivelli, V., Maltoni, B., & Ravaglia, G. (2012). A comparison of frailty indexes for prediction of adverse health outcomes in an elderly cohort. *Archives of gerontology and geriatrics*, *54*(1), 16–20. https://doi.org/10.1016/j.archger.2011.01.007

Fried, L. P., Tangen, C. M., Walston, J., Newman, A. B., Hirsch, C., Gottdiener, J., Seeman, T., Tracy, R., Kop, W. J., Burke, G., McBurnie, M. A., & Cardiovascular Health Study Collaborative Research Group (2001). Frailty in older adults: evidence for a phenotype. *The journals of gerontology. Series A, Biological sciences and medical sciences*, *56*(3), M146–M156. https://doi.org/10.1093/gerona/56.3.m146

García-García, F. J., Carcaillon, L., Fernandez-Tresguerres, J., Alfaro, A., Larrion, J. L., Castillo, C., & Rodriguez-Mañas, L. (2014). A new operational definition of frailty: the Frailty Trait Scale. *Journal of the American Medical Directors Association*, *15*(5), 371.e7–371.e13. https://doi.org/10.1016/j.jamda.2014.01.004

Gilbert, T., Neuburger, J., Kraindler, J., Keeble, E., Smith, P., Ariti, C., Arora, S., Street, A., Parker, S., Roberts, H. C., Bardsley, M., & Conroy, S. (2018). Development and validation of a Hospital Frailty Risk Score focusing on older people in acute care settings using electronic hospital records: an observational study. *Lancet (London, England)*, *391*(10132), 1775–1782. https://doi.org/10.1016/S0140-6736(18)30668-8

Gill, T. M., Gahbauer, E. A., Han, L., & Allore, H. G. (2010). Trajectories of disability in the last year of life. The New England journal of medicine, 362(13), 1173–1180. https://doi.org/10.1056/NEJMoa0909087

Gobbens, R. J., van Assen, M. A., Luijkx, K. G., & Schols, J. M. (2012). The predictive validity of the Tilburg Frailty Indicator: disability, health care utilization, and quality of life in a population at risk. *The Gerontologist*, *52*(5), 619–631. https://doi.org/10.1093/geront/gnr135

Gobbens, R. J., van Assen, M. A., & Schalk, M. J. (2014). The prediction of disability by self-reported physical frailty components of the Tilburg Frailty Indicator (TFI). *Archives of gerontology and geriatrics*, *59*(2), 280–287. https://doi.org/10.1016/j.archger.2014.06.008

Graham, J. E., Snih, S. A., Berges, I. M., Ray, L. A., Markides, K. S., & Ottenbacher, K. J. (2009). Frailty and 10-year mortality in community-living Mexican American older adults. *Gerontology*, *55*(6), 644–651. https://doi.org/10.1159/000235653

Gray, S. L., Anderson, M. L., Hubbard, R. A., LaCroix, A., Crane, P. K., McCormick, W., Bowen, J. D., McCurry, S. M., & Larson, E. B. (2013). Frailty and incident dementia. *The journals of gerontology. Series A, Biological sciences and medical sciences*, *68*(9), 1083–1090. https://doi.org/10.1093/gerona/glt013

Gu, D., Dupre, M. E., Sautter, J., Zhu, H., Liu, Y., & Yi, Z. (2009). Frailty and mortality among Chinese at advanced ages. *The journals of gerontology. Series B, Psychological sciences and social sciences*, *64*(2), 279–289. https://doi.org/10.1093/geronb/gbn009

Gu, D., Yang, F., & Sautter, J. (2016). Socioeconomic status as a moderator between frailty and mortality at old ages. *BMC geriatrics*, *16*, 151. https://doi.org/10.1186/s12877-016-0322-2

Hall, D. E., Arya, S., Schmid, K. K., Blaser, C., Carlson, M. A., Bailey, T. L., Purviance, G., Bockman, T., Lynch, T. G., & Johanning, J. (2017). Development and Initial Validation of the Risk Analysis Index for Measuring Frailty in Surgical Populations. *JAMA surgery*, *152*(2), 175–182. https://doi.org/10.1001/jamasurg.2016.4202

Hall, R. K., Morton, S., Wilson, J., Kim, D. H., Colón-Emeric, C., Scialla, J. J., Platt, A., Ephraim, P. L., Boulware, L. E., & Pendergast, J. (2022). Development of an Administrative Data-Based Frailty Index for Older Adults Receiving Dialysis. *Kidney360*, *3*(9), 1566–1577. https://doi.org/10.34067/KID.0000032022

Hanlon, P., Burton, J. K., Quinn, T. J., Mair, F. S., McAllister, D., Lewsey, J., & Gallacher, K. I. (2023). Prevalence, measurement, and implications of frailty in stroke survivors: An analysis of three global aging cohorts. *International journal of stroke : official journal of the International Stroke Society*, *18*(6), 720–727. https://doi.org/10.1177/17474930231151847

Hastings, S. N., Purser, J. L., Johnson, K. S., Sloane, R. J., & Whitson, H. E. (2008). Frailty predicts some but not all adverse outcomes in older adults discharged from the emergency department. *Journal of the American Geriatrics Society*, *56*(9), 1651–1657. https://doi.org/10.1111/j.1532-5415.2008.01840.x

Hogan, D. B., Freiheit, E. A., Strain, L. A., Patten, S. B., Schmaltz, H. N., Rolfson, D., & Maxwell, C. J. (2012). Comparing frailty measures in their ability to predict adverse outcome among older residents of assisted living. *BMC geriatrics*, *12*, 56. https://doi.org/10.1186/1471-2318-12-56

Jauhari, Y., Gannon, M. R., Dodwell, D., Horgan, K., Clements, K., Medina, J., Tsang, C., Robinson, T., Tang, S. S., Pettengell, R., & Cromwell, D. A. (2020). Construction of the secondary care administrative records frailty (SCARF) index and validation on older women with operable invasive breast cancer in England and Wales: a cohort study. *BMJ open*, *10*(5), e035395. https://doi.org/10.1136/bmjopen-2019-035395

Jones, D., Song, X., Mitnitski, A., & Rockwood, K. (2005). Evaluation of a frailty index based on a comprehensive geriatric assessment in a population based study of elderly Canadians. *Aging clinical and experimental research*, *17*(6), 465–471. https://doi.org/10.1007/BF03327413

Jones, D. M., Song, X., & Rockwood, K. (2004). Operationalizing a frailty index from a standardized comprehensive geriatric assessment. *Journal of the American Geriatrics Society*, *52*(11), 1929–1933. https://doi.org/10.1111/j.1532-5415.2004.52521.x

Joosten, E., Demuynck, M., Detroyer, E., & Milisen, K. (2014). Prevalence of frailty and its ability to predict in hospital delirium, falls, and 6-month mortality in hospitalized older patients. BMC geriatrics, 14, 1. https://doi.org/10.1186/1471-2318-14-1

Jung, H. W., Kim, S. W., Lim, J. Y., Kim, K. W., Jang, H. C., Kim, C. H., & Kim, K. I. (2014). Frailty status can predict further lean body mass decline in older adults. *Journal of the American Geriatrics Society*, *62*(11), 2110–2117. https://doi.org/10.1111/jgs.13107

Kamaruzzaman, S., Ploubidis, G. B., Fletcher, A., & Ebrahim, S. (2010). A reliable measure of frailty for a community dwelling older population. *Health and quality of life outcomes*, *8*, 123. https://doi.org/10.1186/1477-7525-8-123

Khandelwal, D., Goel, A., Kumar, U., Gulati, V., Narang, R., & Dey, A. B. (2012). Frailty is associated with longer hospital stay and increased mortality in hospitalized older patients. The journal of nutrition, health & aging, 16(8), 732–735. https://doi.org/10.1007/s12603-012-0369-5

Kiely, D. K., Cupples, L. A., & Lipsitz, L. A. (2009). Validation and comparison of two frailty indexes: The MOBILIZE Boston Study. *Journal of the American Geriatrics Society*, *57*(9), 1532–1539. https://doi.org/10.1111/j.1532-5415.2009.02394.x

Kim, D. H., Glynn, R. J., Avorn, J., Lipsitz, L. A., Rockwood, K., Pawar, A., & Schneeweiss, S. (2019). Validation of a Claims-Based Frailty Index Against Physical Performance and Adverse Health Outcomes in the Health and Retirement Study. *The journals of gerontology. Series A, Biological sciences and medical sciences*, *74*(8), 1271–1276. https://doi.org/10.1093/gerona/gly197

Kim, D. H., Patorno, E., Pawar, A., Lee, H., Schneeweiss, S., & Glynn, R. J. (2020). Measuring Frailty in Administrative Claims Data: Comparative Performance of Four Claims-Based Frailty Measures in the U.S. Medicare Data. *The journals of gerontology. Series A, Biological sciences and medical sciences*, *75*(6), 1120–1125. https://doi.org/10.1093/gerona/glz224

Kim, D. H., Schneeweiss, S., Glynn, R. J., Lipsitz, L. A., Rockwood, K., & Avorn, J. (2018). Measuring Frailty in Medicare Data: Development and Validation of a Claims-Based Frailty Index. *The journals of gerontology. Series A, Biological sciences and medical sciences*, *73*(7), 980–987. https://doi.org/10.1093/gerona/glx229

Klein, B. E., Klein, R., Knudtson, M. D., & Lee, K. E. (2005). Frailty, morbidity and survival. *Archives of gerontology and geriatrics*, *41*(2), 141–149. https://doi.org/10.1016/j.archger.2005.01.002

Kulminski, A. M., Ukraintseva, S. V., Kulminskaya, I. V., Arbeev, K. G., Land, K., & Yashin, A. I. (2008). Cumulative deficits better characterize susceptibility to death in elderly people than phenotypic frailty: lessons from the Cardiovascular Health Study. *Journal of the American Geriatrics Society*, *56*(5), 898–903. https://doi.org/10.1111/j.1532-5415.2008.01656.x

Kundi, H., Coskun, N., & Yesiltepe, M. (2021). Association of entirely claims-based frailty indices with long-term outcomes in patients with acute myocardial infarction, heart failure, or pneumonia: a nationwide cohort study in Turkey. *The Lancet regional health. Europe*, *10*, 100183. https://doi.org/10.1016/j.lanepe.2021.100183

Le Pogam, M. A., Seematter-Bagnoud, L., Niemi, T., Assouline, D., Gross, N., Trächsel, B., Rousson, V., Peytremann-Bridevaux, I., Burnand, B., & Santos-Eggimann, B. (2022). Development and validation of a knowledge-based score to predict Fried's frailty phenotype across multiple settings using one-year hospital discharge data: The electronic frailty score. *EClinicalMedicine*, *44*, 101260. https://doi.org/10.1016/j.eclinm.2021.101260

Lekan, D. A., Wallace, D. C., McCoy, T. P., Hu, J., Silva, S. G., & Whitson, H. E. (2017). Frailty Assessment in Hospitalized Older Adults Using the Electronic Health Record. *Biological research for nursing*, *19*(2), 213–228. https://doi.org/10.1177/1099800416679730

Liang, Y. D., Xie, Y. B., Du, M. H., Shi, J., Yang, J. F., & Wang, H. (2021). Development and Validation of an Electronic Frailty Index Using Routine Electronic Health Records: An Observational Study From a General Hospital in China. *Frontiers in medicine*, *8*, 731445. https://doi.org/10.3389/fmed.2021.731445

Louis, D. Z., Robeson, M., McAna, J., Maio, V., Keith, S. W., Liu, M., Gonnella, J. S., & Grilli, R. (2014). Predicting risk of hospitalisation or death: a retrospective population-based analysis. *BMJ open*, *4*(9), e005223. https://doi.org/10.1136/bmjopen-2014-005223

Lucicesare, A., Hubbard, R. E., Fallah, N., Forti, P., Searle, S. D., Mitnitski, A., Ravaglia, G., & Rockwood, K. (2010). Comparison of two frailty measures in the Conselice Study of Brain Ageing. *The journal of nutrition, health & aging*, *14*(4), 278–281. https://doi.org/10.1007/s12603-010-0061-6

Ma, S. L., Oyler, J., Glavin, S., Alavi, A., & Vokes, T. (2009). Self-reported frailty is associated with low calcaneal bone mineral density in a multiracial population of community-dwelling elderly. *Osteoporosis international : a journal established as result of cooperation between the European Foundation for Osteoporosis and the National Osteoporosis Foundation of the USA*, *20*(11), 1837–1846. https://doi.org/10.1007/s00198-009-0884-3

Makary, M. A., Segev, D. L., Pronovost, P. J., Syin, D., Bandeen-Roche, K., Patel, P., Takenaga, R., Devgan, L., Holzmueller, C. G., Tian, J., & Fried, L. P. (2010). Frailty as a predictor of surgical outcomes in older patients. *Journal of the American College of Surgeons*, *210*(6), 901–908. https://doi.org/10.1016/j.jamcollsurg.2010.01.028

Mazzaglia, G., Roti, L., Corsini, G., Colombini, A., Maciocco, G., Marchionni, N., Buiatti, E., Ferrucci, L., & Di Bari, M. (2007). Screening of older community-dwelling people at risk for death and hospitalization: the Assistenza Socio-Sanitaria in Italia project. *Journal of the American Geriatrics Society*, *55*(12), 1955–1960. https://doi.org/10.1111/j.1532-5415.2007.01446.x

McIsaac, D. I., Wong, C. A., Huang, A., Moloo, H., & van Walraven, C. (2019). Derivation and Validation of a Generalizable Preoperative Frailty Index Using Population-based Health Administrative Data. *Annals of surgery*, *270*(1), 102–108. https://doi.org/10.1097/SLA.0000000000002769

Mitnitski, A. B., Graham, J. E., Mogilner, A. J., & Rockwood, K. (2002). Frailty, fitness and late-life mortality in relation to chronological and biological age. *BMC geriatrics*, *2*, 1. https://doi.org/10.1186/1471-2318-2-1

Moldovan, M., Khadka, J., Visvanathan, R., Wesselingh, S., & Inacio, M. C. (2020). Using elastic nets to estimate frailty burden from routinely collected national aged care data. *Journal of the American Medical Informatics Association : JAMIA*, *27*(3), 419–428. https://doi.org/10.1093/jamia/ocz210

Morley, J. E., Malmstrom, T. K., & Miller, D. K. (2012). A simple frailty questionnaire (FRAIL) predicts outcomes in middle aged African Americans. *The journal of nutrition, health & aging*, *16*(7), 601–608. https://doi.org/10.1007/s12603-012-0084-2

Nishimura, S., Kumamaru, H., Shoji, S., Nakatani, E., Yamamoto, H., Ichihara, N., Miyachi, Y., Sandhu, A. T., Heidenreich, P. A., Yamauchi, K., Watanabe, M., Miyata, H., & Kohsaka, S. (2022). Assessment of coding-based frailty algorithms for long-term outcome prediction among older people in community settings: a cohort study from the Shizuoka Kokuho Database. *Age and ageing*, *51*(3), afac009. https://doi.org/10.1093/ageing/afac009

O'Caoimh, R., Gao, Y., Svendrovski, A., Healy, E., O'Connell, E., O'Keeffe, G., Cronin, U., O'Herlihy, E., Cornally, N., & Molloy, W. D. (2014). Screening for markers of frailty and perceived risk of adverse outcomes using the Risk Instrument for Screening in the Community (RISC). *BMC geriatrics*, *14*, 104. https://doi.org/10.1186/1471-2318-14-104

Orkaby, A. R., Nussbaum, L., Ho, Y. L., Gagnon, D., Quach, L., Ward, R., Quaden, R., Yaksic, E., Harrington, K., Paik, J. M., Kim, D. H., Wilson, P. W., Gaziano, J. M., Djousse, L., Cho, K., & Driver, J. A. (2019). The Burden of Frailty Among U.S. Veterans and Its Association With Mortality, 2002-2012. *The journals of gerontology. Series A, Biological sciences and medical sciences*, *74*(8), 1257–1264. https://doi.org/10.1093/gerona/gly232

Pajewski, N. M., Lenoir, K., Wells, B. J., Williamson, J. D., & Callahan, K. E. (2019). Frailty Screening Using the Electronic Health Record Within a Medicare Accountable Care Organization. *The journals of gerontology. Series A, Biological sciences and medical sciences*, *74*(11), 1771–1777. https://doi.org/10.1093/gerona/glz017

Pandolfi, P., Collina, N., Marzaroli, P., Stivanello, E., Musti, M. A., Giansante, C., Perlangeli, V., Pizzi, L., De Lisio, S., & Francia, F. (2016). Sviluppo di un modello predittivo di decesso o ricovero d'urgenza per l'individuazione degli anziani fragili [Development of a predictive model of death or urgent hospitalization to identify frail elderly]. *Epidemiologia e prevenzione*, *40*(6), 395–403. https://doi.org/10.19191/EP16.6.P395.119

Pilotto, A., Ferrucci, L., Franceschi, M., D'Ambrosio, L. P., Scarcelli, C., Cascavilla, L., Paris, F., Placentino, G., Seripa, D., Dallapiccola, B., & Leandro, G. (2008). Development and validation of a multidimensional prognostic index for one-year mortality from comprehensive geriatric assessment in hospitalized older patients. *Rejuvenation research*, *11*(1), 151–161. https://doi.org/10.1089/rej.2007.0569

Puts, M. T., Lips, P., & Deeg, D. J. (2005). Static and dynamic measures of frailty predicted decline in performance-based and self-reported physical functioning. *Journal of clinical epidemiology*, *58*(11), 1188–1198. https://doi.org/10.1016/j.jclinepi.2005.03.008

Quach, J., Theou, O., Godin, J., Rockwood, K., & Kehler, D. S. (2022). The impact of cardiovascular health and frailty on mortality for males and females across the life course. *BMC medicine*, *20*(1), 394. https://doi.org/10.1186/s12916-022-02593-w

Ravaglia, G., Forti, P., Lucicesare, A., Pisacane, N., Rietti, E., & Patterson, C. (2008). Development of an easy prognostic score for frailty outcomes in the aged. *Age and ageing*, *37*(2), 161–166. https://doi.org/10.1093/ageing/afm195

Rochat, S., Cumming, R. G., Blyth, F., Creasey, H., Handelsman, D., Le Couteur, D. G., Naganathan, V., Sambrook, P. N., Seibel, M. J., & Waite, L. (2010). Frailty and use of health and community services by community-dwelling older men: the Concord Health and Ageing in Men Project. *Age and ageing*, *39*(2), 228–233. https://doi.org/10.1093/ageing/afp257

Rockwood K. (2005). What would make a definition of frailty successful?. *Age and ageing*, *34*(5), 432–434. https://doi.org/10.1093/ageing/afi146

Rockwood, K., Fox, R. A., Stolee, P., Robertson, D., & Beattie, B. L. (1994). Frailty in elderly people: an evolving concept. *CMAJ : Canadian Medical Association journal = journal de l'Association medicale canadienne*, *150*(4), 489–495.

Rockwood, K., Mitnitski, A., Song, X., Steen, B., & Skoog, I. (2006). Long-term risks of death and institutionalization of elderly people in relation to deficit accumulation at age 70. *Journal of the American Geriatrics Society*, *54*(6), 975–979. https://doi.org/10.1111/j.1532-5415.2006.00738.x

Rockwood, K., Song, X., MacKnight, C., Bergman, H., Hogan, D. B., McDowell, I., & Mitnitski, A. (2005b). A global clinical measure of fitness and frailty in elderly people. *CMAJ : Canadian Medical Association journal = journal de l'Association medicale canadienne*, *173*(5), 489–495. https://doi.org/10.1503/cmaj.050051

Rockwood, K., Stadnyk, K., MacKnight, C., McDowell, I., Hébert, R., & Hogan, D. B. (1999). A brief clinical instrument to classify frailty in elderly people. *Lancet (London, England)*, *353*(9148), 205–206. https://doi.org/10.1016/S0140-6736(98)04402-X

Romero-Ortuno R. (2013). The Frailty Instrument for primary care of the Survey of Health, Ageing and Retirement in Europe predicts mortality similarly to a frailty index based on comprehensive geriatric assessment. *Geriatrics & gerontology international*, *13*(2), 497–504. https://doi.org/10.1111/j.1447-0594.2012.00948.x

Romero-Ortuno, R., & Soraghan, C. (2014). A Frailty Instrument for primary care for those aged 75 years or more: findings from the Survey of Health, Ageing and Retirement in Europe, a longitudinal population-based cohort study (SHARE-FI75+). *BMJ open*, *4*(12), e006645. https://doi.org/10.1136/bmjopen-2014-006645

Rothman, M. D., Leo-Summers, L., & Gill, T. M. (2008). Prognostic significance of potential frailty criteria. *Journal of the American Geriatrics Society*, *56*(12), 2211–2216. https://doi.org/10.1111/j.1532-5415.2008.02008.x

Saliba, D., Elliott, M., Rubenstein, L. Z., Solomon, D. H., Young, R. T., Kamberg, C. J., Roth, C., MacLean, C. H., Shekelle, P. G., Sloss, E. M., & Wenger, N. S. (2001). The Vulnerable Elders Survey: a tool for identifying vulnerable older people in the community. *Journal of the American Geriatrics Society*, *49*(12), 1691–1699. https://doi.org/10.1046/j.1532-5415.2001.49281.x

Samper-Ternent, R., Karmarkar, A., Graham, J., Reistetter, T., & Ottenbacher, K. (2012). Frailty as a predictor of falls in older Mexican Americans. *Journal of aging and health*, *24*(4), 641–653. https://doi.org/10.1177/0898264311428490

Searle, S. D., Mitnitski, A., Gahbauer, E. A., Gill, T. M., & Rockwood, K. (2008). A standard procedure for creating a frailty index. *BMC geriatrics*, *8*, 24. https://doi.org/10.1186/1471-2318-8-24

Segal, J. B., Chang, H.-Y., Du, Y., Walston, J. D., Carlson, M. C., & Varadhan, R. (2017). Development of a Claims-based Frailty Indicator Anchored to a Well-established Frailty Phenotype. *Medical care*, *55*(7), 716–722. https://doi.org/10.1097/MLR.0000000000000729

Segal, J. B., Huang, J., Roth, D. L., & Varadhan, R. (2017b). External validation of the claims-based frailty index in the national health and aging trends study cohort. *American journal of epidemiology*, *186*(6), 745–747. https://doi.org/10.1093/aje/kwx257

Sharma, Y., Horwood, C., Hakendorf, P., Shahi, R., & Thompson, C. (2022). External Validation of the Hospital Frailty-Risk Score in Predicting Clinical Outcomes in Older Heart-Failure Patients in Australia. *Journal of clinical medicine*, *11*(8), 2193. https://doi.org/10.3390/jcm11082193

Shashikumar, S. A., Luke, A. A., Johnston, K. J., & Joynt Maddox, K. E. (2020). Assessment of HF Outcomes Using a Claims-Based Frailty Index. *JACC. Heart failure*, *8*(6), 481–488. https://doi.org/10.1016/j.jchf.2019.12.012

Shen, Y., Wang, Y., Shi, Q., Hou, L., Chen, X., Dong, B., & Hao, Q. (2021). The Electronic Frailty Index is Associated with Increased Infection and All-Cause Mortality Among Older Patients with Primary Lung Cancer: A Cohort Study. *Clinical interventions in aging*, *16*, 1825–1833. https://doi.org/10.2147/CIA.S335172

Shi, J., Song, X., Yu, P., Tang, Z., Mitnitski, A., Fang, X., & Rockwood, K. (2011). Analysis of frailty and survival from late middle age in the Beijing Longitudinal Study of Aging. *BMC geriatrics*, *11*, 17. https://doi.org/10.1186/1471-2318-11-17

Silan, M., Signorin, G., Ferracin, E., Listorti, E., Spadea, T., Costa, G., & Boccuzzo, G. (2022). Construction of a Frailty Indicator with Partially Ordered Sets: A Multiple-Outcome Proposal Based on Administrative Healthcare Data. *Soc Indic Res, 160,* 989–1017. https://doi.org/10.1007/s11205-020-02512-7

Sirola, J., Pitkala, K. H., Tilvis, R. S., Miettinen, T. A., & Strandberg, T. E. (2011). Definition of frailty in older men according to questionnaire data (RAND-36/SF-36): The Helsinki Businessmen Study. *The journal of nutrition, health & aging*, *15*(9), 783–787. https://doi.org/10.1007/s12603-011-0131-4

Solfrizzi, V., Scafato, E., Frisardi, V., Seripa, D., Logroscino, G., Maggi, S., Imbimbo, B. P., Galluzzo, L., Baldereschi, M., Gandin, C., Di Carlo, A., Inzitari, D., Crepaldi, G., Pilotto, A., Panza, F., & Italian Longitudinal Study on Aging Working Group (2013). Frailty syndrome and the risk of vascular dementia: the Italian Longitudinal Study on Aging. *Alzheimer's & dementia : the journal of the Alzheimer's Association*, *9*(2), 113–122. https://doi.org/10.1016/j.jalz.2011.09.223

Soong, J., Poots, A. J., Scott, S., Donald, K., & Bell, D. (2015). Developing and validating a risk prediction model for acute care based on frailty syndromes. *BMJ open*, *5*(10), e008457. https://doi.org/10.1136/bmjopen-2015-008457

Soong, J. T. Y., Kaubryte, J., Liew, D., Peden, C. J., Bottle, A., Bell, D., Cooper, C., & Hopper, A. (2019). Dr Foster global frailty score: an international retrospective observational study developing and validating a risk prediction model for hospitalised older persons from administrative data sets. *BMJ open*, *9*(6), e026759. https://doi.org/10.1136/bmjopen-2018-026759

Song, X., Mitnitski, A., & Rockwood, K. (2010). Prevalence and 10-year outcomes of frailty in older adults in relation to deficit accumulation. *Journal of the American Geriatrics Society*, *58*(4), 681–687. https://doi.org/10.1111/j.1532-5415.2010.02764.x

Song, X., Mitnitski, A., & Rockwood, K. (2011). Nontraditional risk factors combine to predict Alzheimer disease and dementia. *Neurology*, *77*(3), 227–234. https://doi.org/10.1212/WNL.0b013e318225c6bc

St John, P. D., Tyas, S. L., & Montgomery, P. R. (2013). Life satisfaction and frailty in community-based older adults: cross-sectional and prospective analyses. *International psychogeriatrics*, *25*(10), 1709–1716. https://doi.org/10.1017/S1041610213000902

Strawbridge, W. J., Shema, S. J., Balfour, J. L., Higby, H. R., & Kaplan, G. A. (1998). Antecedents of frailty over three decades in an older cohort. *The journals of gerontology. Series B, Psychological sciences and social sciences*, *53*(1), S9–S16. https://doi.org/10.1093/geronb/53b.1.s9

Tarekegn, A., Ricceri, F., Costa, G., Ferracin, E., & Giacobini, M. (2020). Predictive Modeling for Frailty Conditions in Elderly People: Machine Learning Approaches. *JMIR medical informatics*, *8*(6), e16678. https://doi.org/10.2196/16678

Tew, Y. Y., Chan, J. H., Keeling, P., Shenkin, S. D., MacLullich, A., Mills, N. L., Denvir, M. A., & Anand, A. (2021). Predicting readmission and death after hospital discharge: a comparison of conventional frailty measurement with an electronic health record-based score. *Age and ageing*, *50*(5), 1641–1648. https://doi.org/10.1093/ageing/afab043

Theou, O., Brothers, T. D., Mitnitski, A., & Rockwood, K. (2013). Operationalization of frailty using eight commonly used scales and comparison of their ability to predict all-cause mortality. *Journal of the American Geriatrics Society*, *61*(9), 1537–1551. https://doi.org/10.1111/jgs.12420

Tom, S. E., Adachi, J. D., Anderson, F. A., Jr, Boonen, S., Chapurlat, R. D., Compston, J. E., Cooper, C., Gehlbach, S. H., Greenspan, S. L., Hooven, F. H., Nieves, J. W., Pfeilschifter, J., Roux, C., Silverman, S., Wyman, A., LaCroix, A. Z., & GLOW Investigators (2013). Frailty and fracture, disability, and falls: a multiple country study from the global longitudinal study of osteoporosis in women. *Journal of the American Geriatrics Society*, *61*(3), 327–334. https://doi.org/10.1111/jgs.12146

Velanovich, V., Antoine, H., Swartz, A., Peters, D., & Rubinfeld, I. (2013). Accumulating deficits model of frailty and postoperative mortality and morbidity: its application to a national database. *The Journal of surgical research*, *183*(1), 104–110. https://doi.org/10.1016/j.jss.2013.01.021

Vetrano, D. L., Zucchelli, A., Onder, G., Fratiglioni, L., Calderón-Larrañaga, A., Marengoni, A., Marconi, E., Cricelli, I., Lora Aprile, P., Bernabei, R., Cricelli, C., & Lapi, F. (2023). Frailty detection among primary care older patients through the Primary Care Frailty Index (PC-FI). *Scientific reports*, *13*(1), 3543. https://doi.org/10.1038/s41598-023-30350-3

Walker, L., Jamrozik, K., & Wingfield, D. (2005). The Sherbrooke Questionnaire predicts use of emergency services. *Age and ageing*, *34*(3), 233–237. https://doi.org/10.1093/ageing/afi020

Woo, J., Leung, J., & Morley, J. E. (2012). Comparison of frailty indicators based on clinical phenotype and the multiple deficit approach in predicting mortality and physical limitation. *Journal of the American Geriatrics Society*, *60*(8), 1478–1486. https://doi.org/10.1111/j.1532-5415.2012.04074.x

Woods, N. F., LaCroix, A. Z., Gray, S. L., Aragaki, A., Cochrane, B. B., Brunner, R. L., Masaki, K., Murray, A., Newman, A. B., & Women's Health Initiative (2005). Frailty: emergence and consequences in women aged 65 and older in the Women's Health Initiative Observational Study. *Journal of the American Geriatrics Society*, *53*(8), 1321–1330. https://doi.org/10.1111/j.1532-5415.2005.53405.x

Wu, S., Mulcahy, J., Kasper, J. D., Kan, H. J., & Weiner, J. P. (2020). Comparing Survey-Based Frailty Assessment to Medicare Claims in Predicting Health Outcomes and Utilization in Medicare Beneficiaries. *Journal of aging and health*, *32*(7-8), 764–777. https://doi.org/10.1177/0898264319851995

Zhao, F., Tang, B., Liu, X., Weng, W., Wang, B., Wang, Y., Zhang, Z., & Zhang, L. (2022). Development and validation of the geriatric trauma frailty index for geriatric trauma patients based on electronic hospital records. *Age and ageing*, *51*(1), afab186. https://doi.org/10.1093/ageing/afab186

1.2 Health outcome prevalences in 2018

Crude and Standardized prevalences (%)						
Outcome 2018	**Crude prevalences**			**Standardized prevalences**		
	**Female**	**Male**	**Whole population**	**Female**	**Male**	**Ratio F/M** [6]
**Death**	3.67	3.74	3.70	2.95	4.18	0.71
**ER access with highest priority**	1.27	1.37	1.31	1.06	1.48	0.72
**Femur fracture**	0.80	0.32	0.59	0.68	0.36	1.89
**Hospitalization**	15.92	19.37	17.42	15.06	19.74	0.76
**Repeated hospitalization**	5.17	6.90	5.93	4.86	7.02	0.69
**Emergency hospitalization**	14.22	17.30	15.56	13.29	17.69	0.75
**Avoidable hospitalization**	4.53	5.48	4.94	3.89	5.81	0.67
**Disability**	29.02	25.76	27.60	25.66	26.94	0.95
**Dementia**	3.92	2.79	3.43	3.31	3.04	1.09
**Institutionalization**	1.19	0.74	1.00	0.99	0.79	1.25

Table 2: Crude and standardized prevalences (%) of the 10 outcomes observed in 2018. Acronyms: ER: Emergency Room.

[6] Ratio between the female and male standardized prevalences.

1.3 Emerging patterns from correlation and factor analysis

	Rotated factorial pattern		
Outcome	**Factor 1**	**Factor 2**	**Factor 3**
**Death**	0.096	0.108	0.620
**Access to ER-HP**	-0.033	-0.041	0.717
**Femur fracture**	0.368	0.208	-0.422
**Hospitalization**	0.922	-0.057	-0.049
**Repeated hospitalization**	0.728	-0.018	0.073
**Emergency hospitalization**	0.934	-0.045	-0.024
**Disability**	0.195	0.403	0.226
**Dementia**	-0.043	0.748	-0.043
**Institutionalization**	-0.084	0.739	-0.002
**Avoidable hospitalization**	0.396	0.058	0.393

Table 3: Factor patterns (promax rotation) for women in the 65–74 age group. Acronyms: ER-HP: Emergency Room access with Highest Priority.

	**Rotated factorial pattern**		
Outcome	**Factor 1**	**Factor 2**	**Factor 3**
**Death**	0.214	0.306	0.487
**Access to ER-HP**	0.207	0.051	0.599
**Femur fracture**	0.432	0.194	-0.691
**Hospitalization**	0.931	-0.053	-0.015
**Repeated hospitalization**	0.762	-0.081	-0.064
**Emergency hospitalization**	0.938	-0.035	-0.007
**Disability**	0.177	0.557	0.092
**Dementia**	-0.017	0.669	0.082
**Institutionalization**	-0.223	0.782	-0.114
**Avoidable hospitalization**	0.651	0.036	0.269

Table 4: Factor patterns (promax rotation) for women aged 75 and above. Acronyms: ER-HP: Emergency Room access with Highest Priority.

	**Rotated factorial pattern**		
Outcome	**Factor 1**	**Factor 2**	**Factor 3**
**Death**	0.133	0.624	0.116
**Access to ER-HP**	-0.001	0.724	-0.068
**Femur fracture**	0.237	-0.385	0.312
**Hospitalization**	0.935	-0.066	-0.035
**Repeated hospitalization**	0.737	0.063	-0.008
**Emergency hospitalization**	0.943	-0.042	-0.033
**Disability**	0.163	0.335	0.300
**Dementia**	-0.038	-0.070	0.764
**Institutionalization**	-0.092	0.094	0.703
**Avoidable hospitalization**	0.517	0.289	-0.004

Table 5: Factor patterns (promax rotation) for men in the 65–74 age group. Acronyms: ER-HP: Emergency Room access with Highest Priority.

	Rotated factorial pattern		
Outcome	**Factor 1**	**Factor 2**	**Factor 3**
**Death**	0.350	0.431	-0.197
**Access to ER-HP**	0.291	0.207	-0.456
**Femur fracture**	0.165	0.153	0.848
**Hospitalization**	0.927	-0.082	0.065
**Repeated hospitalization**	0.751	-0.064	0.072
**Emergency hospitalization**	0.937	-0.060	0.059
**Disability**	0.229	0.535	0.050
**Dementia**	-0.030	0.706	-0.016
**Institutionalization**	-0.230	0.724	0.123
**Avoidable hospitalization**	0.693	0.103	-0.144

Table 6: Factor patterns (promax rotation) for men aged 75 and above. Acronyms: ER-HP: Emergency Room access with Highest Priority.

1.4 Visualizing outcomes connections

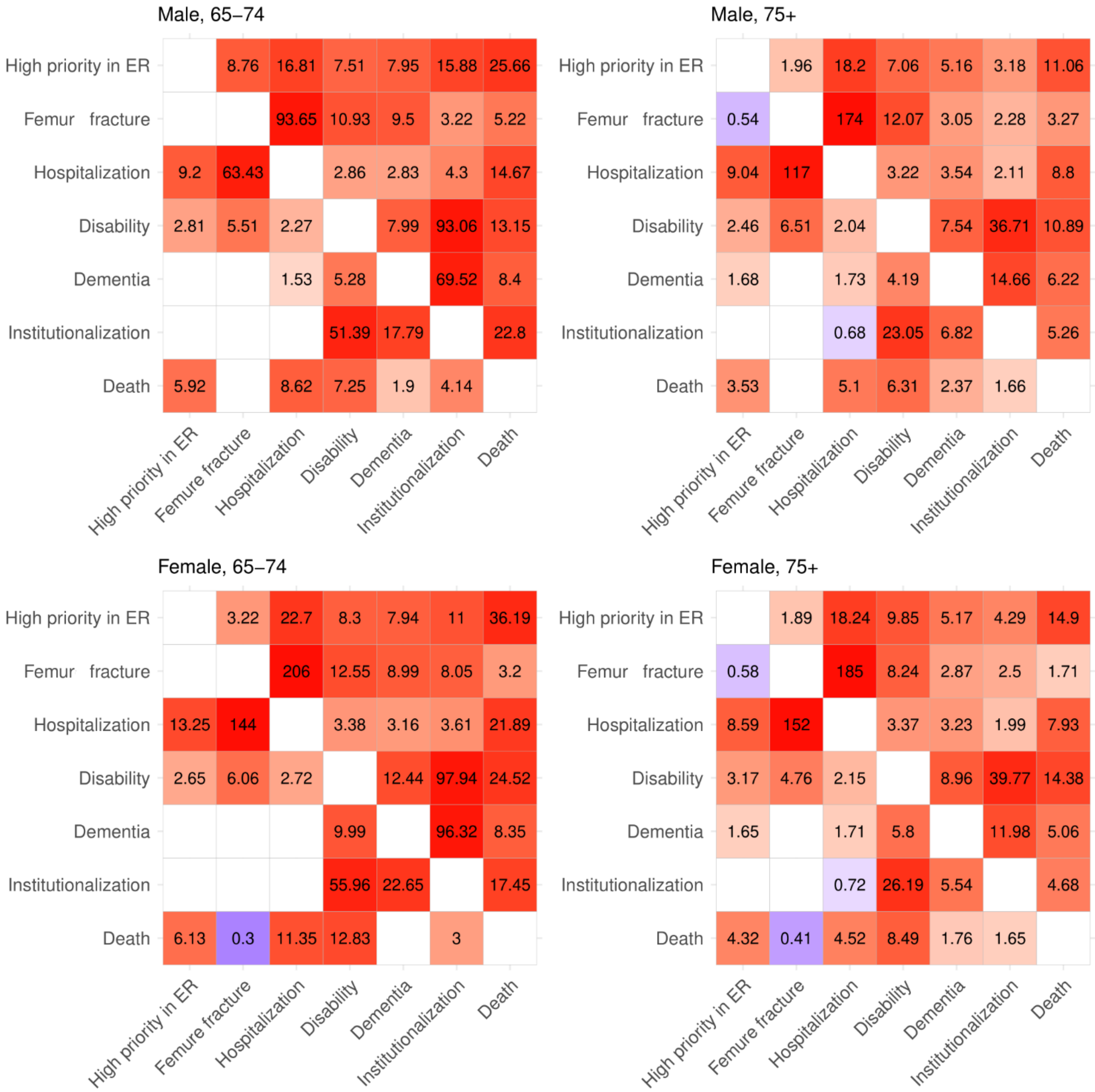

Figure 1: Matrix with the estimated marginal (upper triangular) and conditional (lower triangular) odds ratio conducted separately by sex and age group. The color intensity of the cells is proportional to Yule's Q coefficients, computed from the odds ratio associated with each cell. Red cells indicate positive associations, whereas blue cells represent negative ones, while blank cells indicate conditional independence between the corresponding variables. Acronyms: ER: Emergency Room.
